# Near-Unity Emitting, Widely Tailorable, and Stable Exciton Concentrators Built from Doubly Gradient 2D Semiconductor Nanoplatelets

**DOI:** 10.1021/acsnano.3c05125

**Published:** 2023-08-23

**Authors:** Xiao Liang, Emek G. Durmusoglu, Maria Lunina, Pedro Ludwig Hernandez-Martinez, Vytautas Valuckas, Fei Yan, Yulia Lekina, Vijay Kumar Sharma, Tingting Yin, Son Tung Ha, Ze Xiang Shen, Handong Sun, Arseniy Kuznetsov, Hilmi Volkan Demir

**Affiliations:** † LUMINOUS! Center of Excellence for Semiconductor Lighting and Displays, The Photonics Institute, School of Electrical and Electronic Engineering, Nanyang Technological University, Singapore, 639798, Singapore; ‡ Division of Physics and Applied Physics, School of Physical and Mathematical Sciences, Nanyang Technological University, Singapore, 637371, Singapore; § Interdisciplinary Graduate Program, Nanyang Technological University, Singapore, 637371, Singapore; ∥ Institute of Materials Research and Engineering, A*STAR (Agency for Science, Technology and Research), 2 Fusionopolis Way, #08-03 Innovis, Singapore, 138634, Singapore; ⊥ UNAM—Institute of Materials Science and Nanotechnology, The National Nanotechnology Research Center, Department of Electrical and Electronics Engineering, Department of Physics, Bilkent University, Bilkent, Ankara 06800,Turkey

**Keywords:** semiconductor nanoplatelets, near-unity quantum yield, tailorable electrostatic interactions, high stability, optoelectronics

## Abstract

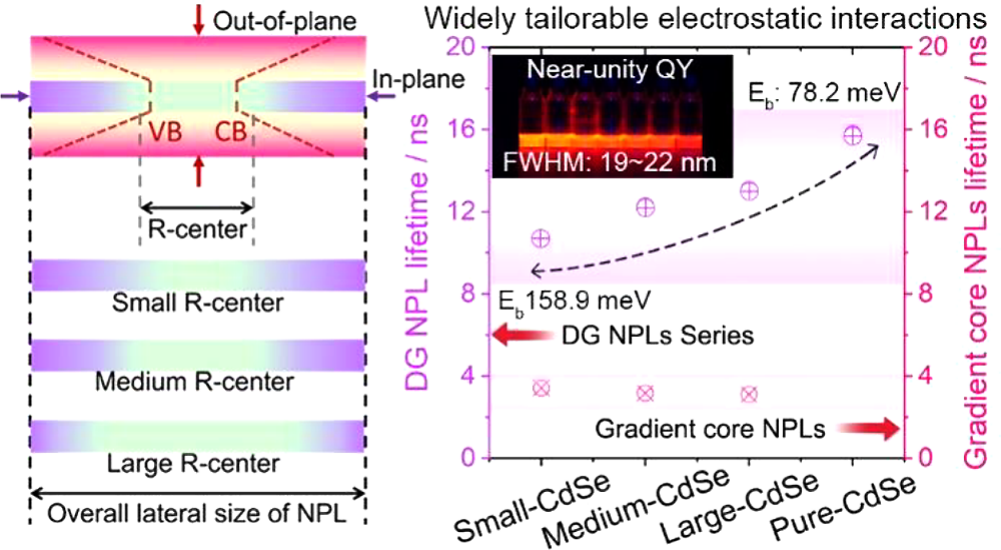

The strength of electrostatic interactions (EIs) between electrons and holes within semiconductor nanocrystals profoundly affects the performance of their optoelectronic systems, and different optoelectronic devices demand distinct EI strength of the active medium. However, achieving a broad range and fine-tuning of the EI strength for specific optoelectronic applications is a daunting challenge, especially in quasi two-dimensional core–shell semiconductor nanoplatelets (NPLs), as the epitaxial growth of the inorganic shell along the direction of the thickness that solely contributes to the quantum confined effect significantly undermines the strength of the EI. Herein we propose and demonstrate a doubly gradient (DG) core–shell architecture of semiconductor NPLs for on-demand tailoring of the EI strength by controlling the localized exciton concentration via in-plane architectural modulation, demonstrated by a wide tuning of radiative recombination rate and exciton binding energy. Moreover, these exciton-concentration-engineered DG NPLs also exhibit a near-unity quantum yield, high photo- and thermal stability, and considerably suppressed self-absorption. As proof-of-concept demonstrations, highly efficient color converters and high-performance light-emitting diodes (external quantum efficiency: 16.9%, maximum luminance: 43,000 cd/m^2^) have been achieved based on the DG NPLs. This work thus provides insights into the development of high-performance colloidal optoelectronic device applications.

## Introduction

Optoelectronic materials, possessing desirable strength and dynamics of electrostatic interactions between electrons and holes,^1−3^ have unlocked the potential for a wide range of cutting-edge optoelectronic applications, such as light-emitting diodes (LEDs),^4−6^ lasers,^7−9^ and quantum information technologies.^10,11^ Within the diverse families of optoelectronic materials, quasi two-dimensional (2D) II–VI semiconductor nanoplatelets (NPLs), also known as colloidal quantum wells, comprised of cadmium chalcogenides (CdX, where X can be Se, S, or a combination thereof) within a zinc blende structure, have attracted increasing attention thanks to their ability to precisely control thickness at the atomic level (typically three, four, or five monolayers),^12^ enabling uniform and strong 1D quantum confinement effects with large absorption cross sections, high exciton binding energies (*E*_b_), and narrow emission line widths.^13−15^ Despite the exceptional properties of atomically flat 2D NPLs, their practical applications have been hindered by their poor stability of quantum efficiency and low tolerance for surface defects, attributed to the unstable nature of the organic ligands used for surface passivation.^16^ To address this challenge, synthetic strategies have been developed, which utilize stable inorganic semiconductors with a wider energy bandgap as surface passivation layer for encapsulating core or core-crown NPLs with shells, enabling a significant improvement in both stability and quantum efficiency.^17,18^ However, the epitaxial growth of the inorganic shell along the direction of the thickness that solely contributes to the quantum confined effect unavoidably undermines the strength of the electrostatic interactions. The aim of independently and selectively tailoring *E*_b_, the energy required to bind an electron and a hole through the electrostatic Coulomb force, while simultaneously maintaining near-unity quantum yield and outstanding stability in existing core–shell (C@S) structures, has been a formidable challenge thus far.^19−21^

The fine-tuning of electrostatic Coulomb force between electrons and holes, known as *E*_b_, is paramount in optoelectronic systems with varying functionalities, as they demand distinct EI strength of the active medium.^1,3^ For instance, judiciously tuning the *E*_b_ in LEDs facilitates exciton generation and recombination according to a sequential electron–hole injection mechanism and boosts electroluminescence (EL) performance.^22,23^ Moreover, strengthened electrostatic interactions enable faster radiative recombination rates in optoelectronic devices, leading to faster switching times and higher data rates in light-based communication.^24,25^ Furthermore, there exists a strong desire for an increased *E*_b_ in the realm of exciton-polariton lasers, as this facilitates robust polariton condensation even at higher temperatures.^26−28^ Therefore, to unlock the full potential of NPLs and facilitate their integration into advanced optoelectronic devices, it is critically important to tackle the challenge of reconciling the conservation of their exceptional stability achieved through thick shells with the fine-tuning of their exciton binding energy.

In addition to the target of on-demand tuning the strength of electrostatic interactions, state-of-the-art C@S NPLs also suffer from significant self-absorption,^29,30^ resulting from the high spatial overlap of exciton generation and recombination, which further limits their efficiency in practical applications. While core–crown–shell (C/C@S) NPLs have partially mitigated this issue by delocalizing electrons to the crown while retaining holes in the core, their reported quantum yield typically falls short of 87% due to nonradiative trap states at the core–crown interface.^31−33^

**Figure 1:**
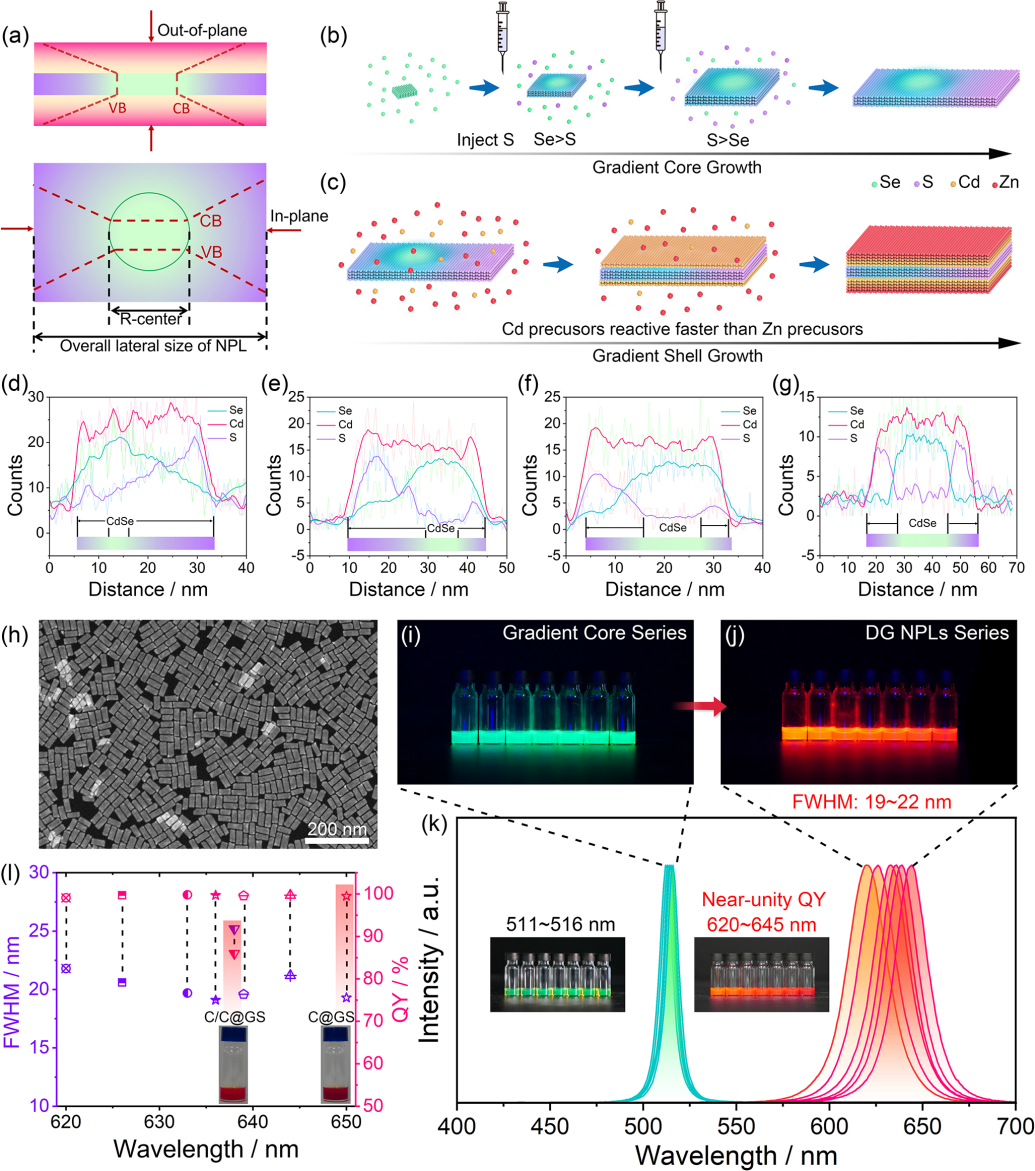
Architectural design, synthetic strategy, and characterizations of DG NPLs. (a) Architectural concept for the proposed doubly gradient NPLs using composition and potential barrier gradients in both out-of-plane (top) and in-plane (bottom) directions. CB and VB refer to the conductive and valence band, respectively. Synthetic methods for the (b) one-pot construction of the gradient core and (c) hot-injection method of gradient shell growth. EDS line-scan results for gradient cores with (d) small, (e) medium, and (f) large size of CdSe seeds and (g) conventional CdSe/CdS core/crown NPLs. (h) Representative HAADF-STEM image of the DG NPLs with a small size of CdSe seed. (i, j) Photographs and (k) emission spectra for the gradient cores and DG NPLs series. (l) fwhm and quantum yields of a DG NPL series in comparison to conventional C@GS and C/C@GS NPLs.

To address the limitations of the state-of-the-art NPLs mentioned above, herein we propose a concept of doubly gradient (DG) architecture with a gradient core–gradient shell configuration, serving as a tailorable exciton concentrator, as illustrated in Figure 1a. Essentially, the proposed architecture operates as a highly efficient light harvester, leveraging on the large absorption coefficient of NPLs, and the designed type-I band offsets from both the in-plane of the gradient core and the out-of-plane of the gradient shell enable high concentrations of the generated excitons toward the recombination center through carrier transfers. Therefore, a large modulation of electrostatic interactions between electrons and holes can be achieved by engineering the localized exciton concentration via tuning the relative lateral dimension of the recombination center (R-center) compared with the overall NPL without altering the shell thickness that is required for high quantum efficiency and high stability. Moreover, this carrier transfer process also spatially separates the exciton generation and recombination, which is beneficial for mitigating the severe self-absorption issue observed in current core–shell NPLs.

To experimentally realize the proposed DG architecture, we construct the gradient core from a CdSe seed with an in-plane compositional gradient transition toward CdS (CdSe/CdSe_*x*_S_1–*x*_), while the gradient shell consists of an out-of-plane compositional gradient transition from CdS to ZnS (Cd_*x*_Zn_1–*x*_S), enabling bidirectional type-I band alignments. Our systematic photophysical studies demonstrate that these DG NPLs function as highly efficient exciton concentrators with superfast and efficient carrier transfer dynamics facilitated by type-I band alignment and smooth interface transitions with fewer defects, leading to a near-unity quantum yield that is unachievable from conventional C/C@S structures. Also, these exciton-concentration-engineered DG NPLs with different CdSe seed sizes demonstrate tunable emission wavelengths ranging from 620 to 645 nm due to the different in-plane spatial confinement, while maintaining near-unity quantum yields and narrow full-width at half-maximum (fwhm) in the range of 19–22 nm. Additionally, significantly reduced self-absorption is achieved in these DG NPLs by up to 59.2% compared to core–gradient shell (C@GS) counterparts. Furthermore, the *E*_b_ and radiative recombination rates are enhanced by up to 103.2% and 54.9%, respectively, compared to C@GS NPLs. Moreover, the higher *E*_b_ as well as the effective passivation of surface defects together contribute to the extraordinary stability of DG NPLs against thermal and UV degradation. Finally, microsized fluorescent patterns composed of these highly efficient and stable DG NPLs, as well as LED devices based on the *E*_b_-tailored DG NPLs, have been shown as proof-of-concept demonstrations. These DG NPLs-based LED devices exhibit an improved external quantum efficiency (EQE) of 16.9% and a maximum luminance of 43,000 cd/m^2^ compared to C@GS NPLs with the same aspect ratio (EQE: 11.2%, maximum luminance: 14,100 cd/m^2^). In summary, these DG NPLs with a near-unity quantum yield, tunable emission wavelength, narrow fwhm, significantly reduced self-absorption, tailorable *E*_b_, and high photo- and thermal stability hold great promise for various practical applications of optoelectronics (color conversion, LEDs, polariton lasers, and so on).

## Results and Discussion

The proposed doubly gradient-structured NPLs were developed from a stepwise synthetic strategy, as depicted in Figure 1b,c. The growth of gradient core NPLs originated from the lateral expansion of four-monolayer (4ML) CdSe seed NPLs. Unlike the synthesis of previously reported core–crown NPLs, where the CdSe core and CdS crown were grown separately,^34,35^ the gradient transition from CdSe to CdS was achieved by introducing controlled amounts of sulfur (S) precursors into the same pot, at a specific rate, after the CdSe seeds had grown to a certain size. As the Se precursor was continually depleted and S was added, the gradient core gradually transformed from CdSe to CdS (Figure 1b). This one-pot synthetic approach enables precise tuning of CdSe seed dimensions over a broad range and facile in-plane architectural engineering of the resultant gradient core heterostructure. Subsequently, to create the gradient shell, the dissimilar reaction rates of Zn and Cd precursors with the S precursors were leveraged,^36,37^ as presented in Figure 1c. The quicker reaction rate between Cd precursors and S generated a primary CdS shell, while the slower participation of Zn precursors occurred at later stages of the reaction, gradually forming the outermost layer of ZnS.^37^ Transmission electron microscopy (TEM) images (Figures S1a–c) reveal the quasi-rectangular shapes of the gradient cores with distinct initiation injection times of S precursors of 100, 200, and 300 s, respectively. The corresponding energy-dispersive X-ray spectroscopy (EDS) line-scan results in Figure 1d–f and Figures S2 and S5 reveal an increased size of CdSe seed from about 3.5 nm to 8 nm and then 12 nm along the long axis of the NPL, denoted as small-, medium-, and large-gradient cores, respectively. Additionally, the EDS line-scan results also demonstrate the compositional gradient and smooth transition from CdSe to CdS, as expected, in all gradient cores. In contrast, the conventional CdSe/CdS core–crown NPLs exhibit a larger CdSe size of approximately 15 nm, with a sharp interface between CdSe and CdS (Figure 1g), consistent with the literature.^34,38^ It is also interesting to note that all the gradient cores feature a rectangular Se-rich side and an irregular wedge-shaped S-rich side (Figure S5b,c), indicating a change in the growth direction of the nanoplatelets from ⟨100⟩ to ⟨110⟩ during the one-pot synthesis. This growth kinetics is relevant to the dynamic changes of the Cd-to-anion ratio during the reaction (Figure S5d), as revealed in previous research.^39^ Following the shell growth, the resulting DG NPL series, indicated as small-, medium-, and large-DG NPLs, built from their corresponding gradient cores (EDS line-scan analysis of individual DG NPLs is provided in Figure S6), exhibit highly homogeneous coating of Cd_*x*_Zn_1–*x*_S shells as well as uniform rectangular morphologies with similar lateral dimensions of length (35 nm) and width (15 nm) (size distribution results are summarized in Figure S8) and an average aspect ratio of around 2.3, under the optimized experimental conditions, as shown from the high-angle annular dark-field scanning TEM (HAADF-STEM) images in Figure 1h and TEM images in Figure S7a–c. Furthermore, to verify the gradient compositional structure along the out-of-plane direction, we carried out EDS mapping characterizations for the cross section of individual DG NPLs using spherical aberration-corrected HAADF-STEM. The results for an individual DG NPL are provided in Figure S9a–c. Figure S9b displays the distributions of each element (Zn, Se, Cd, and S), while Figure S9c reveals the superpositions of EDS signals of different element combinations (Cd+Se, Zn+Se, and Zn+Cd). The above EDS mapping results show that Cd is more concentrated in the gradient cores and the inner part of the shell, whereas Zn is also present in the inner part of the shell but more concentrated in the outer part. Figure S9d presents a high-resolution HAADF-STEM cross-sectional image of an individual DG NPL. The Fourier analysis of lattice periodicity in Figure S9e along the NPL thickness reveals the evolution of lattice constants along the thickness direction. The largest lattice constant of around 0.304 nm is observed in the core region, corresponding to the CdSe recombination center, while gradually decreasing from the inner shell to the outer shell (∼0.28 nm), indicating a gradual compositional transition from CdS to ZnS (Figure S9f–g). Based on the above compositional analysis along both the in-plane and out-of-plane directions, a conclusive demonstration of the doubly gradient architecture proposed in this study is achieved. Additionally, the corresponding schematic band diagrams, along with lateral size dimensions of DG NPLs series, are depicted in Figure S10.

Figure 1i–k and Figure S1d–f show that gradient cores exhibit tunable photoluminescence (PL) and an absorption spectrum, with varying lateral sizes of the CdSe seed. The excitonic peak of the heavy hole continuously blue-shifts with the decrease of CdSe sizes, and the corresponding PL peak shifts from 516 nm to 511 nm. The atomically flat and uniformly thick surface of all gradient cores ensures a consistent quantum confinement effect in the out-of-plane direction. Therefore, the observed blue-shift in the PL peak is mainly attributed to an additional quantum confinement effect in the in-plane direction, which becomes more prominent with decreasing CdSe seed size.^40,41^ Upon completion of the growth of the gradient shell, the DG NPLs series can cover a wider tuning range of 620–645 nm (Figure 1k), demonstrating that the in-plane quantum confinement effect becomes more prominent as the out-of-plane quantum confinement effect relatively weakens, allowing for wider spectral tunability. Notably, all DG NPL series maintain a near-unity quantum yield and an extremely narrow fwhm of 19–22 nm (Figure 1l), attributed to the high-quality shell growth, as evidenced by the TEM and HAADF-STEM images (Figure S11), which significantly reduces the inhomogeneous emission broadening by suppressing the core/shell exciton–phonon coupling.^18,20,42^ It is noteworthy that by reducing the size of CdSe even more, the emission peak can be blue-shifted even further, but it also leads to a broader fwhm of the final product (Figure S12a). Additionally, the emission line widths can also be influenced by changing the Cd/Zn ratio in the shell precursors (Figure S12b), which is consistent with previous reports.^20^ Moreover, we also synthesized conventional C@GS and core/crown@gradient shell (C/C@GS) structured NPL samples (Figures S13 and S14) to compare with the proposed doubly gradient architecture. We find that C@GS NPLs can achieve near-unity quantum efficiency only after sufficient shell growth, losing the ability to control the emission wavelength. Repeating the synthesis of C/C@GS structured NPLs results in a maximum quantum yield of around 85% with an fwhm of approximately 25 nm (Figure 1l), consistent with the literature.^33,38^ Therefore, the DG NPLs demonstrate superior fluorescence properties and wider spectral tunability compared with state-of-the-art C@GS and C/C@GS NPLs.

**Figure 2:**
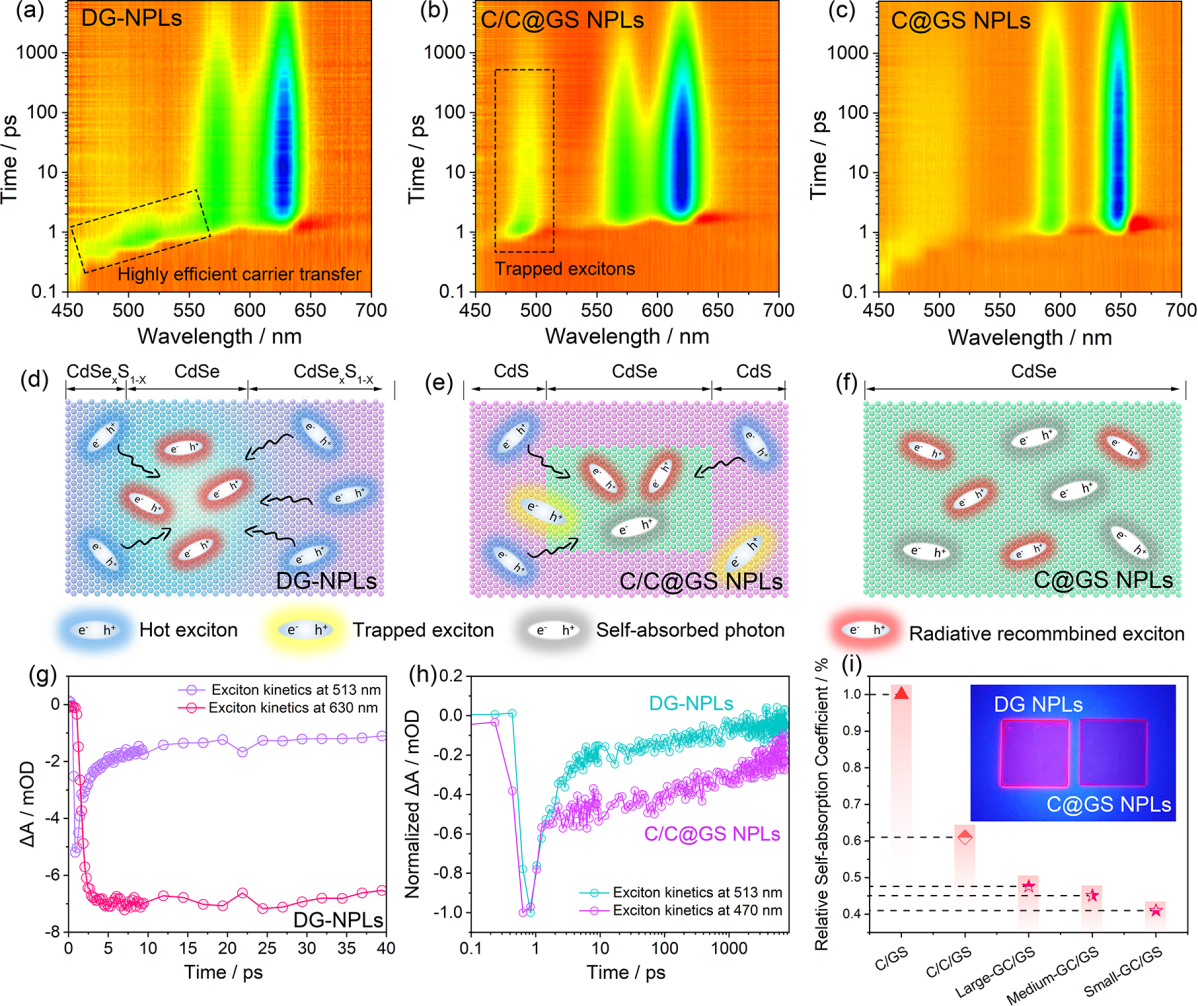
Comparative exciton dynamics of DG NPLs, C/C@GS NPLs, and C@GS NPLs. Transient absorption spectroscopies of (a) DG NPLs in comparison to (b) C/C@GS and (e) C@GS NPLs. Schematic illustrations of carrier transfer processes in (d) DG NPLs, (e) C/C@GS NPLs, and (f) C@GS NPLs. (g) Exciton kinetics of DG NPLs at 513 and 630 nm. (h) Exciton kinetics of DG NPLs and C/C@GS NPLs at 513 and 470 nm, respectively. (i) Relative self-absorption coefficient of the DG NPL series in comparison to C@GS and C/C@GS NPLs. Inset shows the photographs of DG NPL and C@GS NPL films deposited on quartz substrates under uniform exposure of 405 nm excitation light.

To investigate the photophysical properties of DG NPLs in comparison with C@GS and C/C@GS NPLs, we utilized transient absorption (TA) spectroscopy to study their carrier dynamics. Our results, presented in Figure 2a–c and Figure S15, demonstrate that, upon photoexcitation, DG NPLs exhibit an ultrafast and highly efficient in-plane carrier transfer process, with the bleach signal experiencing a continuous and rapid red-shift from 450 nm (S-rich region) to 630 nm (CdSe region) in the first ∼10 ps (Figure S16a–c). Also, during this time, the intensity of the bleach signal in the S-rich region decreases by more than 90%, accompanied by a concomitant increase in the CdSe region (Figure 2g). The driving force for the concentration of excitons toward the CdSe center stems from the energy offsets in the conduction (0.18 eV) and valence bands (0.42 eV) of CdSe and CdS for 4ML NPLs,^43^ as well as their Coulombic interactions, given that holes experience a larger band offset and tend to localize more in the CdSe region. Apart from the TA spectroscopy, we performed wave function calculations to illustrate the probability density of the electrons and the holes within medium-DG NPLs, as presented in Figure S17a. In Figure S17b, we depict the overlap of electron and hole probabilities along the in-plane direction. The simulation results indicate that, although electrons exhibit greater delocalization compared to holes, the majority of their wave functions still overlap within the CdSe center. Leveraging the large absorption coefficient of NPLs, the DG NPLs take full advantage of this ultrafast and highly efficient in-plane carrier transfer process to effectively enrich the generated excitons to the R-center (CdSe seed). This step is pivotal in guaranteeing a near-unity quantum yield and validates the expected exceptional exciton enrichment ability of the DG-NPL, as illustrated in Figure 2d. Since the DG NPL series possess similar overall lateral sizes (Figure S8), a smaller-sized CdSe recombination center will ultimately experience a higher localized exciton concentration via this highly efficient carrier transfer process, enabling a large modulation of the strength of electrostatic interactions between electrons and holes. In contrast, the intensity of the bleach signal in the crown region of the C/C@GS NPL only decreases by 50% in the first 10 ps, followed by a much slower decay tail (Figure 2b and 2h and Figure S16d). Further verifications of the time-resolved PL decay spectra using a streak camera (Figure S18) demonstrate there were no satellite emissions except for the emission from the CdSe recombination center, indicating that the slow decay process of exciton dynamics in Figure 2h can be attributed to the trapped carriers at the core/crown (CdSe/CdS) interface during the relaxation process (Figure 2e), resulting in a less efficient quantum yield. Moreover, the in-plane carrier transfer process in DG-NPLs spatially separates exciton generation and recombination (Figure 2d), resulting in significantly reduced self-absorption compared to C@GS NPLs, where exciton generation and recombination significantly overlap in space, leading to severe self-absorption (Figure 2c and 2f). Notably, the calculated relative self-absorption coefficient indicates that DG NPLs experience up to 59.2% less self-absorption than C@GS NPLs, as summarized in Figure 2i (details of the calculation are provided in Figures S19 and S20). The corresponding thin films of DG-NPLs and C@GS NPLs (insets in Figure 2i) reveal that the DG-NPL film exhibits much brighter edges at the quartz substrate under the same photoexcitation conditions, indicating less energy loss during waveguide propagation due to self-absorption.

**Figure 3:**
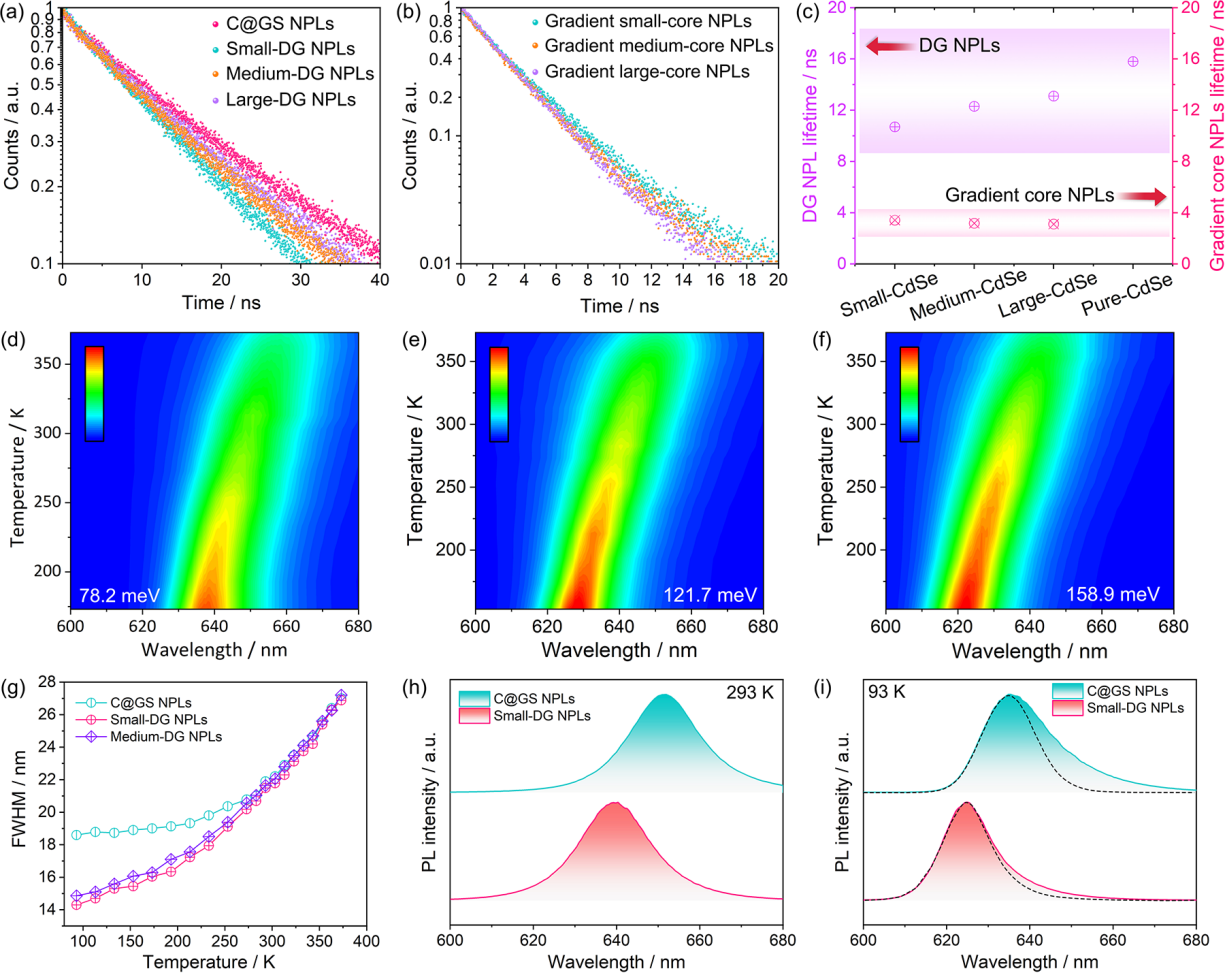
Widely tailorable electrostatic interactions in “defect-free” DG NPLs. (a) PL decays of the DG NPL series in comparison to C@GS NPLs. (b) PL decays of the gradient core series. (c) Summarized radiative fluorescent lifetime of DG NPLs, C@GS NPLs, and gradient core NPLs. (d–f) 2D contour plots of temperature-dependent PL spectra of C@GS NPLs and DG NPLs with (e) medium and (f) small sizes of CdSe seeds, denoted as medium- and small-DG NPLs, respectively. (g) Temperature dependence of fwhm’s of small-DG NPLs, medium-DG NPLs, and C@GS NPLs. Comparative PL spectra of small-DG NPLs and C@GS NPLs at (h) 293 K and (i) 93 K, respectively.

By exploiting the inherent carrier transfer mechanism of DG NPLs with type-I band alignments, it is possible to tune the exciton concentration within the R-center over a wide range by manipulating the relative lateral size of the center compared to the overall NPL, affording a substantial modulation of the strength of electrostatic Coulomb interactions between electrons and holes, which can be deduced by studying their radiative recombination behavior. To compare the radiative behavior of these various exciton-concentration-engineered DG NPLs, we conducted time-resolved PL measurements and analyzed their PL decay kinetics in comparison with conventional C@GS NPLs, as presented in Figure 3a. All samples display near-monoexponential PL decay, in excellent agreement with their near-unity quantum yields. However, DG NPLs exhibit a marked reduction in radiative lifetime, dropping from 15.8 ns (for C@GS NPLs) to 10.2 ns (for small-DG NPLs). This represents a 54.9% increase in the radiative recombination rate, attributed to the strengthened electrostatic Coulomb interactions between electrons and holes within the recombination center, compared to C@GS NPLs. Furthermore, we find that the variations in CdSe size within the gradient core have a relatively small impact on the PL decay kinetics and display an opposite trend to that of the DG-NPL structure (Figure 3b). Specifically, as the CdSe size decreases, the radiative lifetime of the gradient core exhibited an increasing trend. This is primarily due to the strong one-dimensional quantum confinement effect originating from the ultrathin thickness (1.2 nm) of the gradient core squeezing the electron wave function along the out-of-plane direction and planarly extending it throughout the entire gradient core NPLs, as the conduction band offset between CdSe and CdSe_*x*_S_1–*x*_ is relatively small, reducing the spatial overlap between the wave functions of electrons and holes.^38,44,45^ As a result, the lifetime increases with decreasing CdSe size in all gradient core series. However, the in-plane delocalization effect of electrons in core–shell-structured NPLs is mitigated compared to gradient cores, as the quantum confinement of core–shell-structured NPLs in the out-of-plane direction is considerably compromised. It is worth noting that the competition between electron delocalization and in-plane spatial confinement also exists within the DG NPL structure. Specifically, as CdSe size is further reduced, a reversed increase in lifetime is observed in the DG NPL (Figure S21). However, our experimental results show that the largely relaxed quantum confinement in the out-of-plane direction within the core–shell structure still provides ample room for the engineering of in-plane exciton concentration to tune their electrostatic interactions and radiative behavior (Figure 3c).

To further quantify the strength of electrostatic Coulomb interactions between electrons and holes, we conducted a temperature-dependent PL analysis, as depicted in Figure 3d–f. By fitting integrated PL intensity as a function of the temperature (Figure S22), the calculated *E*_b_ values for C@GS NPLs, medium-DG NPLs, and small-DG NPLs were 78.2, 121.7, and 158.9 meV, respectively, achieving a large enhancement of the *E*_b_ by up to 103.2% in the exciton-concentration-engineered DG NPLs. These findings show the highly tunable electrostatic interactions enabled by the exciton-enrichment strategy, which are crucial for achieving high-performance LED devices and ultra-low-threshold exciton-polariton lasing.^22,23,26−28^ Furthermore, in the temperature-dependent PL results, we observe that the fwhm of both C@GS NPLs and DG NPLs, as shown in Figure 3g, shows a consistent trend above ∼270 K, ascribed to the stronger exciton–phonon coupling with increasing temperature.^46−48^ However, below 270 K, the fwhm of C@GS NPLs decreases at a significantly slower rate compared to that of DG NPLs. Comparison of the PL spectra of samples at 293 and 93 K in Figure 3h,i reveals that C@GS NPLs exhibit more asymmetric PL spectra with a pronounced long tail on the lower photon-energy side at low temperatures. Previous studies have attributed the emission on the low-energy side to trap states with longer PL lifetime, as the thermal energy is insufficient to pass the energy barrier of the traps at lower temperatures.^48^ In contrast, the emission from the trap states in DG NPLs at low temperatures is greatly suppressed. These results strongly suggest that the doubly gradient architecture with a smooth interface transition greatly reduces the defects within the NPLs and yields a closer-to-perfect crystal structure.

**Figure 4:**
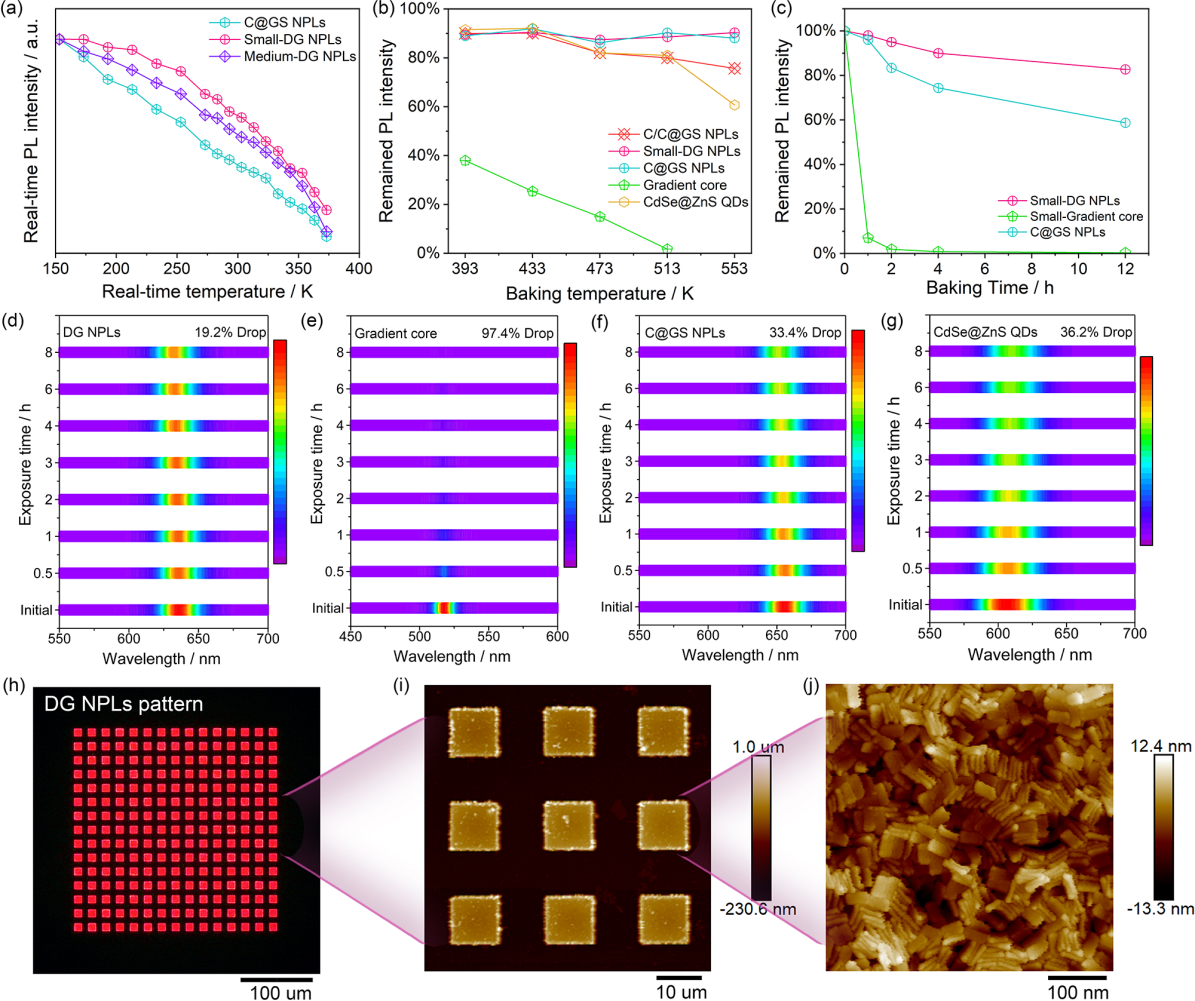
Comparative thermal and photostabilities and conceptual demonstration of DG NPLs as high-resolution microcolor converters. (a) Temperature-dependent real-time PL intensities of small-DG NPLs, medium-DG NPLs, and C@GS NPLs. (b) Thermal stabilities of structurally different emitters under inert conditions. (c) Long-term thermal stabilities of DG NPLs, gradient core NPLs, and C@GS NPLs at 393 K. Long-term photostabilities of (d) DG NPLs, (e) gradient core NPLs, (f) C@GS NPLs, and CdSe@ZnS QDs. (h) Fluorescent image of DG NPL arrays composed of 10-μm-sized pixels. (i) Large-scale and (j) zoomed-in AFM image of DG NPL pixels.

As building blocks for practical optoelectronic applications, thermally and photostable nanocrystals are highly desirable for long-term use and commercial deployment.^18,49,50^ Therefore, we systematically carried out comparative investigations on the photo- and thermal stability of these DG NPLs. Temperature-dependent real-time PL intensities in Figure 4a show that the thermally induced exciton dissociation effects in DG NPLs have weakened during heating thanks to their higher *E*_b_, resulting in a stronger real-time PL intensity level compared to C@GS NPLs. Furthermore, under an inert environment, both DG NPLs and C@GS NPLs can maintain ∼90% of their PL intensity after being kept at 553 K for 10 min and then cooled to room temperature (Figure 4b and Figure S23). In contrast, the PL of gradient cores is almost completely quenched after being subjected to the same treatment at 513 K, highlighting the crucial role played by the inorganic shells in maintaining the stability. Additionally, the PL of C/C@GS NPLs and CdSe/ZnS quantum dots (QDs) decreases by 26% and 43% after being subjected to the same conditions at 553 K, respectively, underscoring the effectiveness of smooth interface transitions in minimizing lattice defects caused by lattice expansion and contraction during heat cycles. DG NPLs also exhibit superior performance compared to C@GS NPLs and gradient cores after long-term heat treatment in the air for 12 h at 353 and 393 K (Figure 4c and Figures S24 and S25), by preserving over 96% and 83% of their initial PL intensity, respectively, which can be attributed to the effective passivation of defects from both the basal plane and edges in the doubly gradient architecture.

In addition to thermal stability, photostability is also a critical factor, particularly in color conversion applications where prolonged exposure to blue light is often necessary. Such a condition tends to result in a decline in PL due to surface oxidation.^51,52^ Results in Figure 4d–g demonstrate that DG NPLs can maintain more than 80.0% of their PL intensity even after 12 h of high-power UV exposure under ambient air conditions. In contrast, C@GS NPLs and QDs can only maintain 67.3% and 66.0% of their initial PL intensity, respectively. On the other hand, gradient core NPLs experience a nearly complete quenching after a similar 12-hour UV exposure in the air. The above results indicate that DG NPLs exhibit superior tolerance to surface defects thanks to their higher *E*_b_ and the highly efficient carrier transfer process toward the CdSe recombination center driven by the gradient band offset along the in-plane direction. These DG NPLs, with a near-unity quantum yield, significantly reduced self-absorption, and exceptional photo- and thermal stability, exhibit great potential for energy-efficient color conversion applications. As a proof of concept shown in Figure 4h–j, we have demonstrated their ability to be patterned into microsized (10 μm) arrays with a large thickness of 500 nm of each pixel (Figure S26), highlighting the potentials of these DG NPLs as highly effective color converters for ultra-high-resolution micro-LED displays.

**Figure 5:**
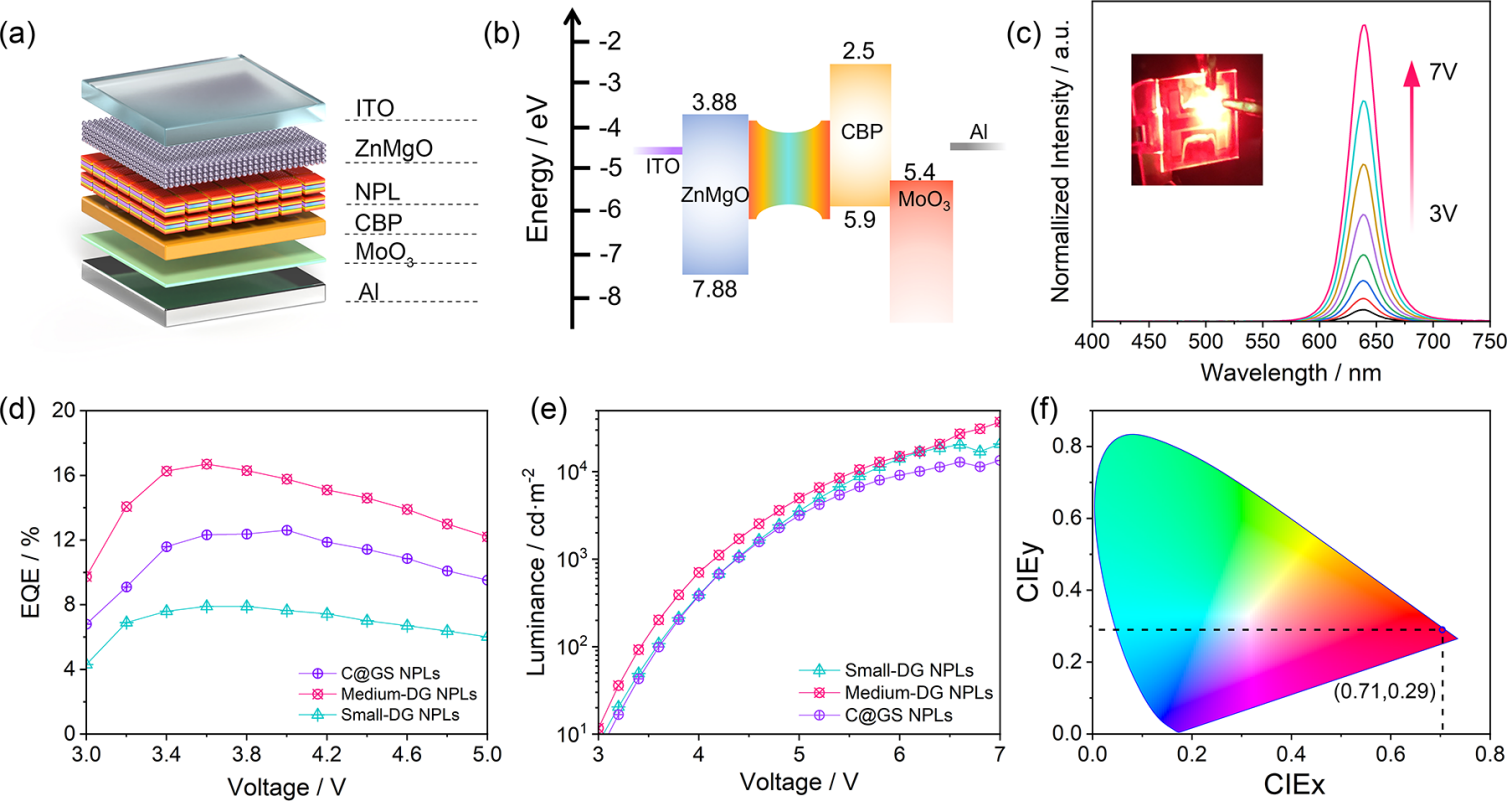
High-performance electroluminescence from exciton-concentration-engineered DG NPLs. (a) Schematic illustration of the LED stacking structure. (b) Energy diagram of the medium-DG NPLs-based LEDs. (e) EL spectra of the DG NPLs-based LEDs operating at various applied voltages. (d) EQE and (e) luminance of LED devices built from small-DG NPLs, medium-DG NPLs, and C@GS NPLs. (f) Chromaticity diagram and position of the PL signal resulting from the medium-DG NPLs.

Furthermore, in LED devices, the appropriate interaction between electrons and holes induced by charge confinement is advantageous for promoting exciton generation and accelerating radiative recombination while suppressing nonradiative Auger recombination, which can be achieved by meticulously tuning the exciton binding energy.^53,54^ Therefore, the highly tunable *E*_b_ in exciton-concentration-engineered DG NPLs holds promise for realizing high-performance LED devices. Here, we chose NPLs with different *E*_b_ and fabricated three comparative devices using an inverted LED device structure, denoted as small-DG-NPL-LED, medium-DG-NPL-LED, and C@GS-NPL-LED, respectively, as shown in Figure 5a,b. Moreover, since the aspect ratio of NPLs plays a crucial role in film smoothness and affects LED device performance,^55^ the three different NPLs we used here possess a similar aspect ratio of around 2.3. Results in Figure 5d,e show that the medium-DG-NPL-LED exhibits the highest external quantum efficiency of 16.9% and the highest brightness of 43,000 cd/m^2^, with a 5.7% improvement in EQE and much enhanced maximum brightness compared to C@GS-NPL-LED under the same conditions (current density and luminance variations at different applied voltages are provided in Figure S27). Furthermore, this device also exhibits highly stable electroluminescence spectra at different voltages (Figure 5c) along with an impressive color purity, as evidenced by the CIE coordinates of (0.71,0.29) (Figure 5f). Besides, it reaches the peak quantum efficiency at a lower voltage of ∼3.5 V, while the C@GS-NPL-LED reaches its peak at 4.1 V (Figure 5d). To further understand the reasons for the results from different LED devices, we conducted comparative power-dependent TA spectroscopy measurements for medium-DG NPLs, small-DG NPLs, and C@GS NPLs, respectively, and the results are summarized in Figures S28 and 29. For DG NPLs, as the size of CdSe becomes smaller, both the radiative (Figure S29a) and nonradiative Auger processes (Figure S29b) accelerate compared to C@GS NPLs due to increased electrostatic interactions within the CdSe recombination center. The weight of the fast process, summarized in Figure S29c, indicates that the contribution of the nonradiative Auger recombination process is comparable between medium-DG NPLs (30.2%) and C@GS NPLs (27.6%) under a low level of pump fluence. Consequently, the faster radiative recombination process, combined with the less pronounced nonradiative Auger recombination process, results in a higher EQE (16.9%) in our medium-DG-NPL-LED devices at a lower driving voltage of 3.5 V. In contrast, in C@GS NPLs, due to fewer carrier confinements and a slower radiative recombination rate, the radiative carrier recombination process in these C@GS-NPL-LED devices is less efficient, and the maximum EQE occurs at a higher driving voltage of 4.1 V. As for small-DG-NPL-LED devices, despite the fast radiative recombination, the faster and prominent Auger-related nonradiative recombination (Figure S29c) becomes dominant even at low driving voltages, leading to a substantially reduced EQE value. The above results demonstrate that the ability to adjust *E*_b_ as needed and the significantly reduced self-absorption coefficient achieved through the DG architecture allow for exciton-concentration-engineered DG NPLs to enhance the performance of LED devices in both EQE and brightness.

## Conclusions

In summary, this study introduces a doubly gradient architecture for semiconductor NPLs, offering an on-demand, widely adjustable exciton concentration to regulate EI through in-plane structural engineering as well as an ultrafast and highly efficient carrier transfer process. The resulting DG NPLs show customizable exciton binding energies and radiative recombination rates, with the highest *E*_b_ and fastest radiative recombination rates being 103.2% and 54.9% greater than those of state-of-the-art C@GS NPLs, respectively. Moreover, the spatial separation of exciton generation and recombination in the doubly gradient NPL architecture also leads to a notably reduced self-absorption of up to 59.2%. Furthermore, the DG NPLs exhibit near-unity quantum yields, adjustable emission wavelength from 620 to 645 nm, narrow fwhm in the range of 19–22 nm, and impressive stability. The above flexible features of DG NPLs make them highly customizable for specific optoelectronic applications. As proof-of-concept demonstrations, we present their potential applications as highly efficient pixelated color converters and for enabling high-performance LED devices with an EQE of 16.9% and a maximum luminance of 43,000 cd/m^2^. This study demonstrates an approach to overcoming the impediments of advancing colloidal II–VI semiconductor NPLs for various optoelectronic applications and achieving their high-performance colloidal optoelectronic systems.

## Methods

### Optical Characterizations

The UV–vis absorption and emission spectra of the NPLs were measured with a Shimadzu UV-1800 spectrophotometer and a Shimadzu RF-5301 PC spectro-fluorophotometer, respectively. To measure the quantum yields, the samples were excited using a 405 nm laser within an integrating sphere, and the data were acquired through an Ocean Optics S4000 spectrometer. To perform time-resolved photoluminescence (TRPL) measurements, a time-correlated single-photon counting (TCSPC) system was employed, utilizing a PicoHarp 300 instrument that can achieve a time resolution as low as 4 ps, which delivered laser pulses at an 80 MHz repetition rate. The setup comprised a driver module (PDL-800 series) that drove a picosecond pulsed laser with a photon energy output of 3.31 eV (375 nm) and a fast photomultiplier tube (Hamamatsu H5783 series) capable of resolving lifetimes at the picosecond level. A streak camera (Optronis) system with a temporal resolution of ∼50 ps was also employed for TRPL measurement to resolve the PL spectra evolution at different decay intervals. To examine the carrier dynamics of the samples, transient absorption (TA) spectroscopy was performed in transmission mode using a Helios setup (Ultrafast Systems LLC) with chirp correction. The pump beam spot size was approximately 50 μm, and the white light continuum probe beam (ranging from 400 to 800 nm) was produced from a 3 mm sapphire crystal by utilizing an 800 nm pulse from the regenerative amplifier. The ultraviolet–visible region detector (CMOS sensor) was utilized to collect the probe beam after it had passed through the sample. Temperature-dependent PL (TDPL) measurements were conducted with a Linkam THMS600 temperature microscope stage. This system allows the sample to be heated and frozen in the range from −78 to 873 K. Cooling was achieved using liquid nitrogen, while electrical heating was used for the temperature increase. The sample was measured in the range of 373 to 153 K with decreasing temperature. The sample was placed in a hermetically sealed chamber and kept under a nitrogen atmosphere to prevent condensation of the water from the air. The stage was assembled with a Witec confocal Raman microscope.

### Structural Characterizations and Elemental Analysis

For structural and elemental characterizations of the synthesized NPLs, a JEOL 2100F transmission electron microscope and a JEM-ARM200F spherical-arbitration-corrected TEM were employed. The JEOL 2100F was operated at 200 kV in the HAADF-STEM configuration and equipped with an EDS detector. JEM-ARM200F was also operated at 200 kV with a STEM resolution of 0.078 nm. Additionally, it was equipped with an EDS detector capable of generating elemental maps at an atomic resolution.

### Atomic Force Microscopy (AFM) Measurement

The AFM measurement was performed using the scan-assist mode with 512 resolutions by a Bruker Dimension Icon scanning probe microscope (Bruker 18 Co., Germany).

### LED Device Characterization

A PR705 spectrometer was used to record the CIE coordinates and EL spectra. Measurements of the current density and luminance as a function of the applied voltage were performed by using a computer-controlled source meter consisting of a programmable Agilent B2902A source meter and a Konica-Minolta LS-110 luminance meter. All measurements were conducted in air at room temperature. The EQE values of the fabricated LED devices were calculated from the obtained luminance, current density, and EL spectrum.

## Supporting Information Available

Detailed information about materials synthesis, device fabrication, additional STEM/TEM/EDS line-scan characterizations, absorption/PL spectra, size distributions, schematic band diagrams of DG NPLs, TA spectra, numerical simulation results of electron and hole wave functions, time-resolved PL spectra, calculations of the relative self-absorption coefficient, integrated PL intensity as a function of temperature, stability measurements, AFM characterizations, applied voltage dependent current density and luminance of the samples, and power-dependent TA characterizations (PDF)
