# Supplementary material for: Near-Unity Emitting, Widely Tailorable, and Stable Exciton Concentrators Built from Doubly Gradient 2D Semiconductor Nanoplatelets
Source: ACS Nano. 2023 Aug 23;17(20):19981–92. doi: 10.1021/acsnano.3c05125 (PMC10603796; doi:10.1021/acsnano.3c05125)
Supplement: Supplementary file 1 — nn3c05125_si_001.pdf [file nn3c05125_si_001.pdf]

Supplementary Information: Near-Unity Emitting, Widely Tailorable and Stable Exciton Concentrators Built from Doubly Gradient 2D Semiconductor Nanoplatelets

*Xiao Liang,[1] Emek G. Durmusoglu,[1,2] Maria Lunina,[3] Pedro Ludwig Hernandez-Martinez,[1] Vytautas Valuckas,[4] Fei Yan,[1] Yulia Lekina,[2] Vijay Kumar Sharma,[1] Tingting Yin,[2] Son Tung Ha,[4] Ze Xiang Shen,[2] Handong Sun,[2] Arseniy Kuznetsov,[4] and Hilmi Volkan Demir[1,2,5]**

[1]LUMINOUS! Center of Excellence for Semiconductor Lighting and Displays, The Photonics Institute, School of Electrical and Electronic Engineering, Nanyang Technological University, Singapore, 639798, Singapore

[2]Division of Physics and Applied Physics, School of Physical and Mathematical Sciences, Nanyang Technological University, Singapore, 637371, Singapore

[3]Interdisciplinary Graduate Program, Nanyang Technological University, Singapore, 637371 Singapore

[4]Institute of Materials Research and Engineering, A*STAR (Agency for Science, Technology and Research), 2 Fusionopolis Way, #08-03 Innovis, 138634, Singapore

[5]UNAM—Institute of Materials Science and Nanotechnology, The National Nanotechnology Research Center, Department of Electrical and Electronics Engineering, Department of Physics, Bilkent University, Bilkent, Ankara, 06800, Turkey

E-mail: hvdemir@ntu.edu.sg

Chemicals

Cadmium oxide (CdO, 99.9%), cadmium acetate dihydrate ($Cd(OAc)_2·2H_2O$, >98%), cadmium nitrate tetrahydrate ($Cd(NO_3)_2·4H_2O$, 99.997%, trace metals basis), sodium myristate (⩾99%), selenium (Se, 99.99%, trace metals basis), sulfur (S, 99,998%, trace metals basis), zinc acetate ($Zn(OAc)_2$, 99.99%, trace metals basis), znic acetate dihydrate ($Zn(OAc)_2·2H_2O$, 99.999%, trace metal basis), 1-octanethiol (⩾98.5%), trioctylamine (TOA, 98%), magnesium acetate tetrahydrate ($Mg(OAc)_2·4H_2O$, ⩾99%), tetramethylammonium hydroxide pentahydrate (TMAH, ⩾97%), ethanolamine (⩾99%), dimethyl sulfoxide (DMSO, ⩾99.9%) 1-octadecene (ODE, technical-grade), oleic acid (OA, 90%), oleylamine (OAm, 70%), and trioctylphoshine (TOP, 90%) were purchased from Sigma-Aldrich. Methanol, hexane, butanol, acetone, toluene, and ethanol were obtained from Merck Millipore and used without any further purification.

Preparation of Cadmium myristate precursors

The synthesis of cadmium myristate ($Cd(Myr)_2$) was carried out by following a previously reported method. [1] Firstly, 1.23 g of $Cd(NO_3)_2·4H_2O$ and 3.13 g of sodium myristate was separately dissolved in 40 mL and 250 mL of methanol, respectively. Once both chemicals had completely dissolved, the solutions were mixed and stirred vigorously for 1 h, resulting in the precipitation of $Cd(Myr)_2$. The precipitate was separated by centrifugation and then washed by redispersing in methanol to remove any excess or unreacted precursors. The above washing step was repeated at least three times, and the precipitate was dried at 50 °C under vacuum overnight before use.

Preparation of 0.1 M S-ODE precursors

0.1 M S-ODE solution was prepared by fully dissolving 32 mg of S in 10 mL of ODE using ultrasonic for 30 min.

Preparation of anisotropic growth solution for CdS crown

A mixture of 480 mg of $Cd(OAc)_2 \cdot 2H_2O$, 340 μL of OA, and 2 mL of ODE in a 10 mL three-neck flask was sonicated for 30 min at room temperature. The solution was then heated to 160 °C under continuous stirring in ambient atmosphere until the formation of a whitish colored homogeneous gel. After that, the above cadmium precursors were combined with 3 mL of 0.1 M S-ODE precursors for the CdS crown growth.

Preparation of 0.25 M $Zn(OA)_2$ precursors

0.92 g of $Zn(OAc)_2$ and 2.8 g of OA were mixed with 20 mL of TOA in a 100 mL three-neck flask. The above mixture was then degassed under room temperature for 30 min. Subsequently, the temperature was heated to 200 °C for 20 min under N_2 protection until the mixture became clear. The resulting solution was heated to 100 °C before use.

Synthesis of $CdSe/CdSe_xS_{1-x}$ 4ML Gradient cores

Typically, a mixture of 170 mg of $Cd(Myr)_2$, 12 mg of Se powder, and 15 mL of ODE was introduced into a 50 mL three-neck flask. After 30 min of degassing at room temperature, the atmosphere was replaced with N_2 and the temperature was set to 230 °C. At approximately 195 °C, 80 mg of $Cd(OAc)_2 \cdot 2H_2O$ was rapidly added, and the gradient transition from CdSe to CdS was initiated by introducing a total 1.5 mL of 0.1 M S-ODE precursors at a steady rate into the same flask after the CdSe seeds had reached a certain size. The resulting solution was heated at 240 °C for 8 min before being cooled to room temperature. At 150 °C, 1 mL of OA was introduced, followed by the addition of 10 mL of hexane at 60°C. The resulting gradient cores were then precipitated by adding 5 mL of ethanol and separated by centrifugating at 4000 rpm for 10 min. Finally, the as-made gradient cores were washed once more before being ultimately redispersed in 5 mL of hexane for further use.

Synthesis of quasi-squarish and quasi-rectangular 4ML CdSe cores

4ML CdSe cores with quasi-squarish shape were synthesized in accordance with established protocols with slight modifications. [2] Specifically, 170 mg of $Cd(Myr)_2$ and 12 mg of Se powder were mixed with 15 mL of ODE in a 50 mL three-necked flask. Following a 20 min degassing period at room temperature, the solution was purged with N_2 and heated to 240 °C. At approximately 195 °C, 80 mg of $Cd(OAc)_2{\cdot}2H_2O$ was swiftly added. After 10 min growth, the solution was cooled to room temperature. During the cooling process, 1 mL of OA was introduced at 150 °C, followed by the addition of 10 mL of hexane at 60°C. The resulting CdSe 4ML cores were precipitated by introducing 5 mL of ethanol and separated by centrifugating at 4000 rpm for 10 min. Finally, the as-made NPLs were washed once more before being ultimately redispersed in 5 mL of hexane for further use. For the synthesis of CdSe 4ML core NPLs possessing a quasi-rectangular shape (aspect ratio of ~2), $Cd(OAc)_2{\cdot}2H_2O$ was substituted with dried $Cd(OAc)_2$, with all remaining procedures identical.

Synthesis of CdSe/CdS 4ML core/crown NPLs

CdSe/CdS 4ML core/crown NPLs were synthesized according to an establish protocol. [3] Typically, 1 mL of 4ML CdSe hexane dispersion, along with 5 mL of ODE and 100 μL of OA, was mixed within a 50 mL three-neck flask. The above solution was then degassed at 80 °C to fully eliminate hexane and air. Subsequently, the temperature was raised to 240 °C under N_2 protection. After that, the anisotropic CdS crown growth mixture was slowly introduced into the flask at a rate of 8 mL/h. Then, the resultant mixture was further annealed for 5 min at 240 °C. The solution was then cooled to room temperature, and the CdSe/CdS core/crown products were precipitated by using ethanol. Ultimately, the precipitated NPLs were dissolved in hexane for further use.

Gradient shell growth for synthesizing DG NPLs, C/C@GS NPLs, and C@GS NPLs

A mixture of 23 mg of $Cd(OAc)_2 \cdot 2H_2O$, 55 mg of $Zn(OAc)_2$, 1 mL of OA, and 10 mL of ODE was combined in a 50 mL three-neck flask, which was subjected to vacuum at room temperature for 20 min, followed by switching the atmosphere to N_2. Subsequently, the mixture was heated to 200 °C and maintained at this temperature for 30 min until a clear solution was obtained. The temperature was then lowered to 60 °C, and either 1 mL of CdSe/$CdSe_xS_{1-x}$ 4ML gradient cores, CdSe/CdS 4ML core/crown NPLs or CdSe 4ML cores in hexane was introduced. Then, the mixture was degassed for 45 min under vacuum to eliminate residual hexane. After that, N_2 flow was initiated through the Schlenk line. The solution was then heated to 300 °C, and 1 mL of degassed OAm was added at 90 °C. A solution of 4 mL of 0.1 M 1-octanethiol in ODE was employed as the S-source, and the injection was carried out at a rate of 8 mL/h using a syringe pump, starting at 165 °C and continuing until the precursor was fully injected. After that, the solution was maintained at this temperature for 40 min of annealing, before the reaction was quenched using a water bath. At 60 °C, 10 mL of hexane was added, and the as-synthesized NPLs were precipitated using 10 mL of ethanol and washed twice. Finally, the precipitated NPLs were redispersed in hexane or toluene for further use.

Synthesis of CdSe@ZnS QDs

CdSe@ZnS QDs were synthesized with slight modifications to a previously reported method. [4] Specifically, a mixture of 0.206 g of CdO and 1.8 g of OA was dissolved into 40 mL of TOA and then degassed. The mixture was heated to 150 °C with rapid stirring, followed by further heating to 300 °C under N_2 flow. At 300 °C, 0.2 mL of 2.0 M Se-TOP was rapidly injected. After 90 s, 1.2 mmol of 1-octanethiol dissolved in TOA (210 μL in 6 mL) was injected at a rate of 1 mL/min using a syringe pump, and the reaction was allowed to proceed for 40 min. Subsequently, the 0.25 M Zn $(OA)_2$ precursor solution was injected into the Cd-containing reaction medium at a rate of 2 mL/min. After

that, 6.4 mmol of 1-octanethiol dissolved in TOA (1.12 mL in 6 mL) was added at a rate of 1 mL/min using a syringe pump. The reaction was allowed to proceed for a total of 2 h. After completion of the reaction, the solution was cooled to room temperature and the organic sludge was removed *via* centrifugation at 5000 rpm for 10 min. Ethanol was then added to the product solution until an opaque flocculant appeared, and the resulting QDs were separated by further centrifugation. Finally, the precipitates were dispersed in toluene/hexane for further use.

Synthesis of $Zn_{0.95}Mg_{0.05}O$ nanocrystals

$Zn_{0.95}Mg_{0.05}O$ nanocrystals were synthesized using a previously reported protocol.[5] Initially, 2.85 mol of $Zn(OAc)_2{\cdot}2H_2O$ and 0.15 mol of $Mg(OAc)_2{\cdot}4H_2O$ were dissolved in 30 mL of DMSO. Next, 1g of TMAH was dissolved in 10 mL of ethanol, and the resulting solution was dropwisely introduced to the above Zn/Mg solution and stirred for 1 h under ambient conditions. After completion of the injection, the solution was stirred at ambient temperature for another 1 h. Subsequently, 250 μL of ethanolamine was added to the solution, and the mixture was stirred for an additional 30 min. Excessive ethyl acetate or acetone were added until the solution became turbid. Finally, the resulting precipitates were separated by centrifuging at 6000 rpm for 3 min and then redispersed in 15 mL of butanol for further use.

Fabrication of florescent NPLs pattern

A direct deep patterning technique was utilized to create fluorescent NPLs patterns with slight modifications.[6] Basically, a sacrificial layer of PMMA (Poly(methylmethacrylate)) resist was firstly spin-coated on a quartz slide at 2400 rpm and baked on a hotplate at 180 °C for 3 min, resulting in a 400 nm PMMA film. Subsequently, a thin layer of electrification dissipating material (ESPACER 300AX01) was spin-coated on top of the resist at 1500 rpm. The resulting sample was then patterned

using e-beam lithography (Elionix ELS-7000) with acceleration voltage of 100 kV and probe current of 500 pA, developed in MIBK:IPA 1:3 solution for 70 s. After that, the NPLs were deposited onto the substrate with the patterned PMMA sacrificial layer *via* spin-coating. Finally, the PMMA sacrificial layer was lifted-off by ultrasonication in acetone for 20 s, leaving only the developed NPL patterns on the quartz substrate.

LED device fabrication

$Zn_{0.95}Mg_{0.05}O$ nanocrystals, used as electron transport layer, were deposited on a ITO-coated glass substrate *via* spin-coating, followed by baking at 120 °C for 30 min in the glovebox. After that, NPLs were deposited onto the above substrate by spin-coating at 2000 rpm for 60 s. Subsequently, the resulting substrate were transferred to a vacuum thermal evaporation chamber for the deposition of CBP, MoO_3 and Al layers at a base pressure of 2.5×10^{-4} Pa, with the thicknesses of each layer being accurately controlled using quartz crystal oscillators. Finally, under N_2 atmosphere, the devices were encapsulated immediately using epoxy glue and glass slides.

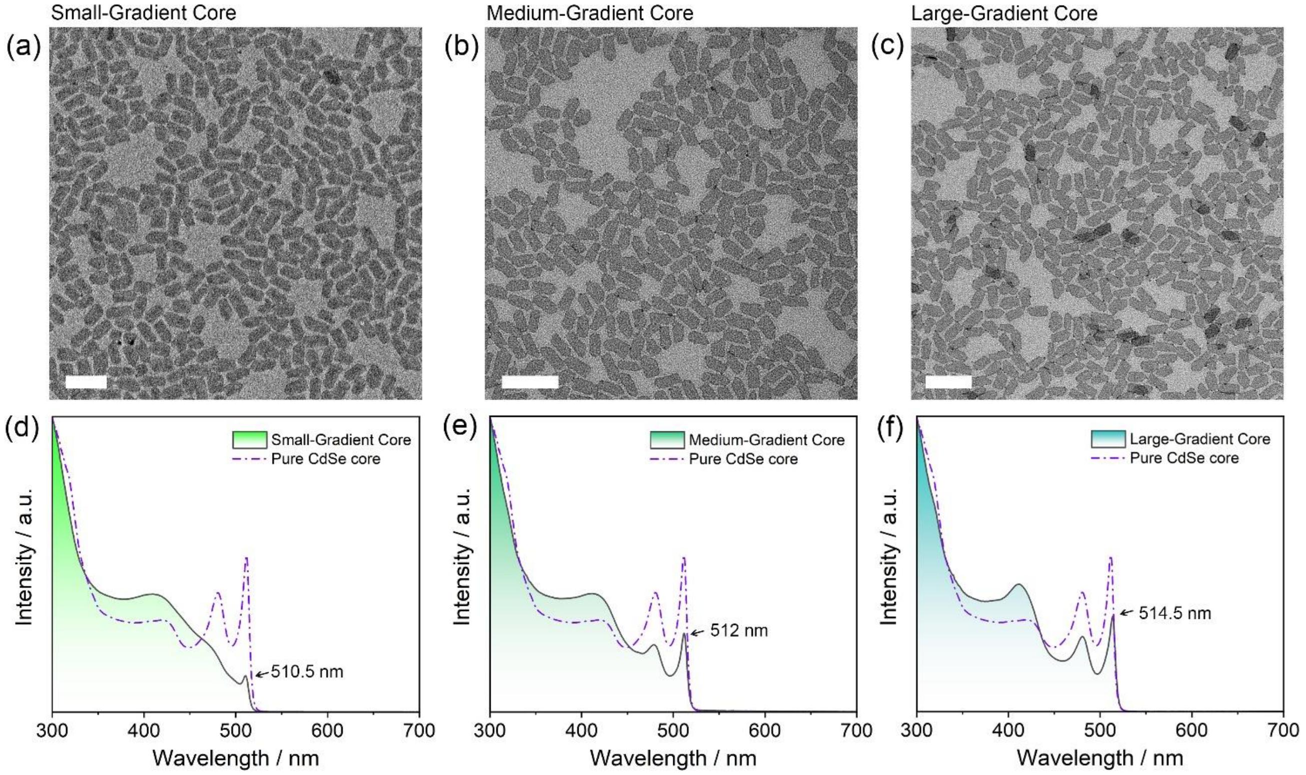

Figure S1. TEM images of the (a) small-, (b) medium-, and (c) large-gradient cores (scale bar: 50 nm). Steady-state absorption spectra of the (d) small-, (e) medium-, and (f) large-gradient cores with the start injection time of S precursors at 100, 200, and 300 s, respectively. The dash-dotted lines represent the normalized absorption spectra of the pure 4ML CdSe cores. It can be seen that the earlier injection of S precursors leads to a gradual decrease in the relative intensity of the excitonic absorption peak of CdSe. This observation indicates a reduction in the area of CdSe recombination center relative to the entire gradient core area, which is consistent with the results obtained from the EDS line-scan, demonstrating that the relative proportion of CdSe (recombination center) to the entire gradient core can be effectively controlled by regulating the initiation injection time of S precursors.

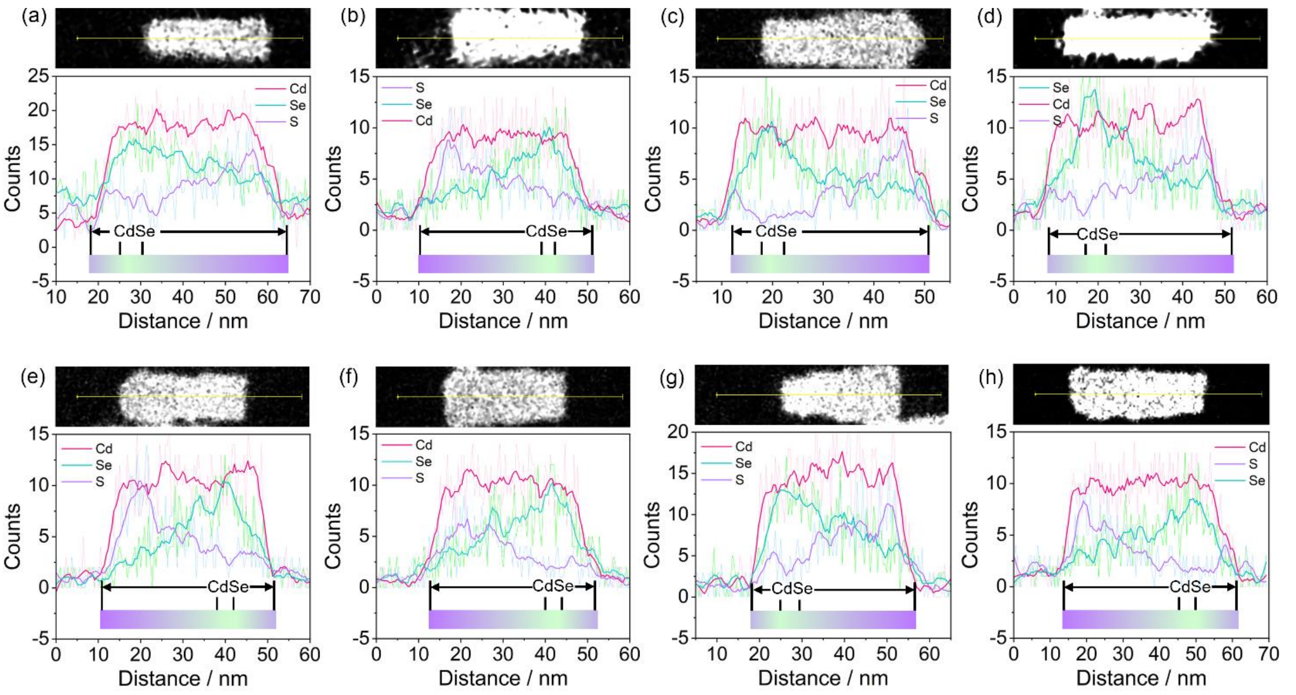

Figure S2. (a-h) Statistic EDS line-scan analysis of 8 individual gradient cores with small CdSe size.

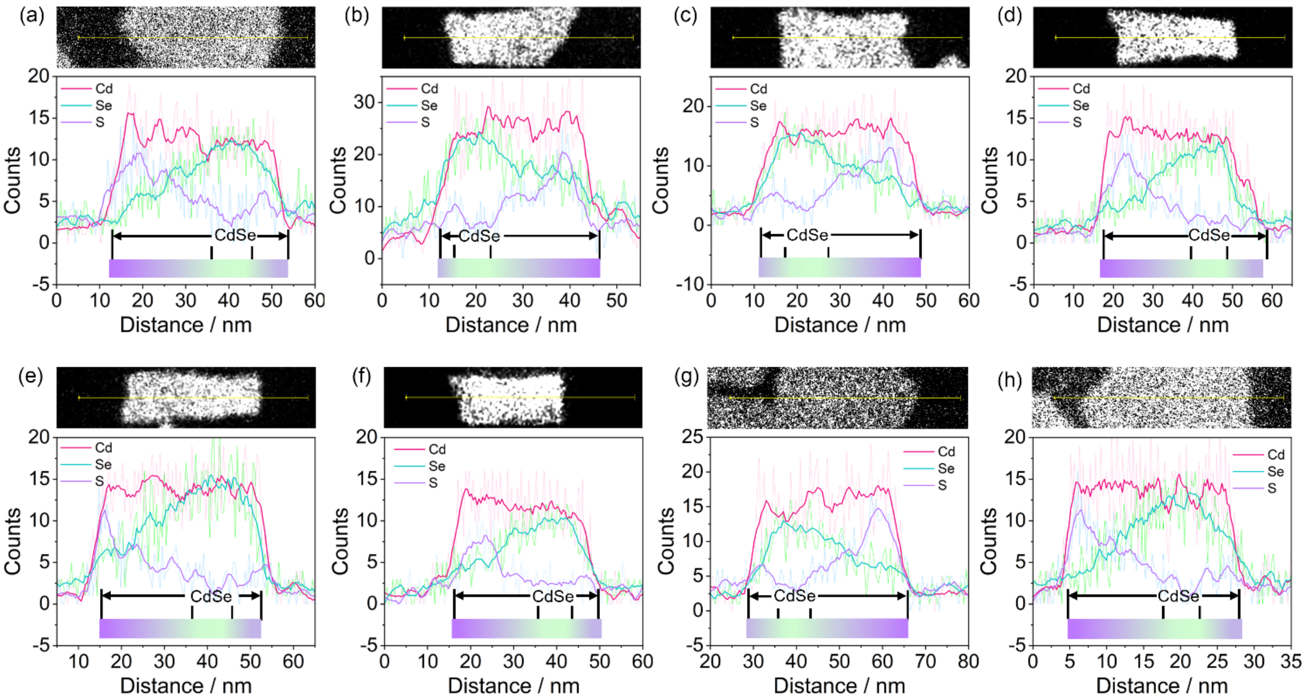

Figure S3. (a-h) Statistic EDS line-scan analysis of 8 individual gradient cores with medium CdSe size.

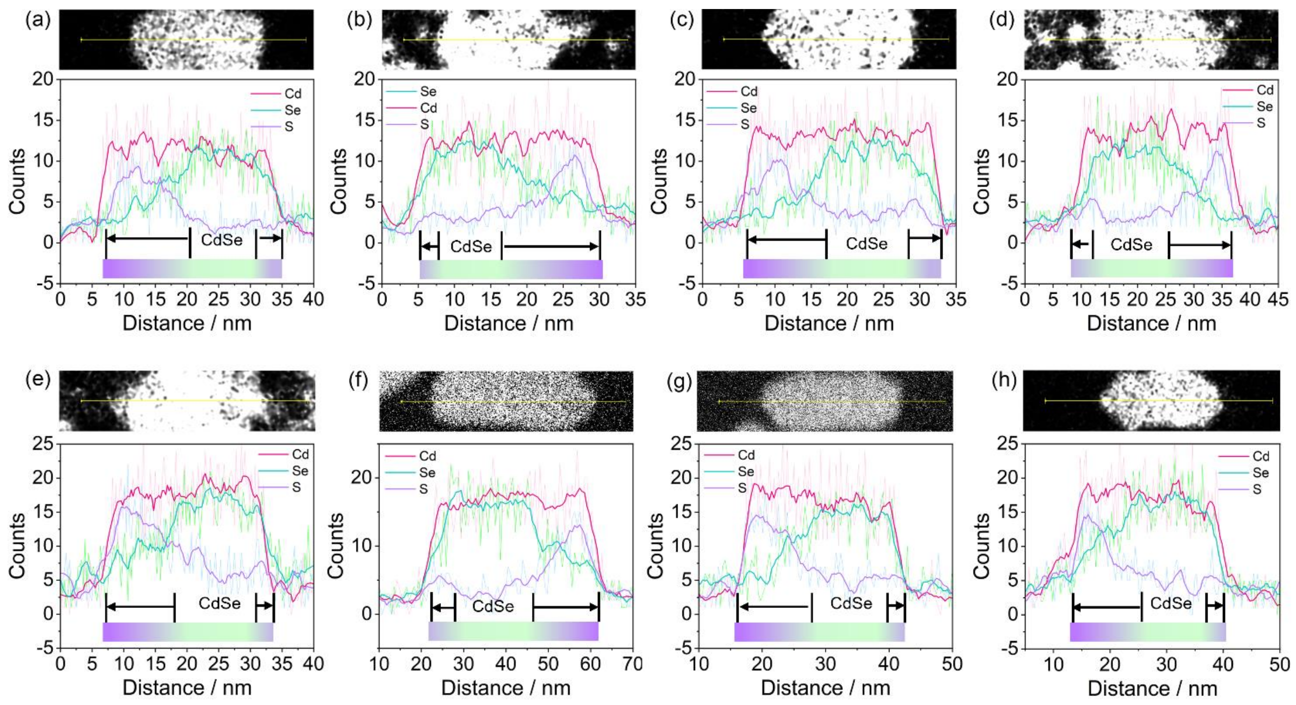

Figure S4. (a-h) Statistic EDS line-scan analysis of 8 individual gradient cores with large CdSe size.

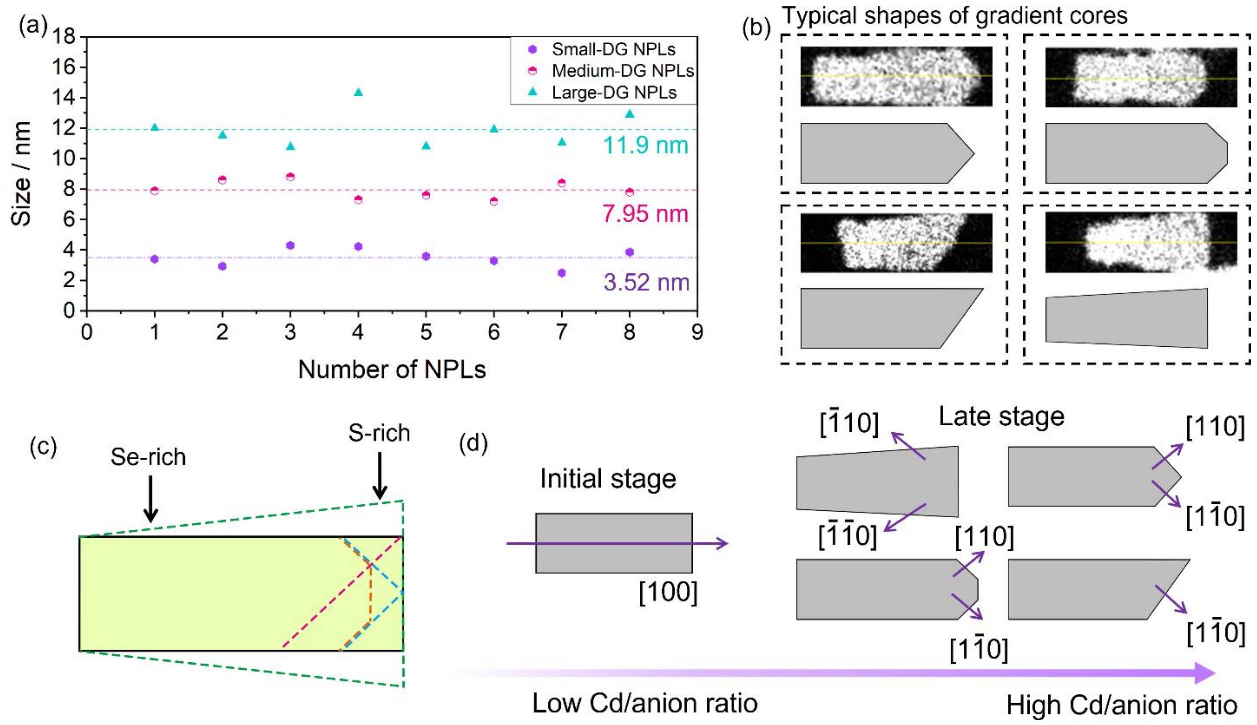

Figure S5. (a) Size distributions and averaged sizes of gradient cores series obtained from EDS line-scan analysis. (b) Typical morphologies of the gradient cores series. (c) Schematic illustrations of the typical shapes of the gradient core series, featuring a rectangular Se-rich side and a wedge-shaped S-rich side. (d) Schematic illustrations of the Cd/anion ratio-determined growth kinetics of the gradient cores.

According to the findings from a previous study on the growth kinetics of 4 monolayer (ML) CdSe

NPLs[7], the growth direction of an individual 4 ML CdSe NPL is governed by the cation-to-anion (Cd -to-Se) precursor ratio. Specifically, a low Cd-to-Se ratio results in a preferred <100> growth direction, leading to the formation of rectangularly shaped NPLs, whereas a high Cd-to-Se ratio results in a preferred <110> growth direction, leading to the formation of irregular wedge-shaped or squarish NPLs.

The statistical results from our gradient cores align well with the growth kinetics mechanism determined by the cation-to-anion ratio. In the initial stage of the gradient core growth, the relatively low Cd-to-anion (Se) ratio leads to a preferred <100> growth direction, resulting in the formation of a Se-rich side with a rectangular shape (**Figure S5d**). Although the S precursor is continuously supplemented during the gradient core growth, the amount of Cd precursors is twice as high as the total amount of anions (Se + S) precursors. As a result, in the late stage of the gradient core growth, the increasing Cd-to-anion ratio gradually shifts the preferred growth direction from <100> to <110>, leading to the formation of an S-rich side with an irregular wedged shape (**Figure S5d**).

The statistical TEM/EDS analysis, combined with insights from the previous study on growth kinetics, explains the elemental distributions of Se and S in our gradient cores, as well as the unique shapes of the gradient cores in this study. Our experimental results may also serve as a reference for controlling the morphologies of gradient cores and offer insights for optimizing relevant optoelectronic properties in future studies.

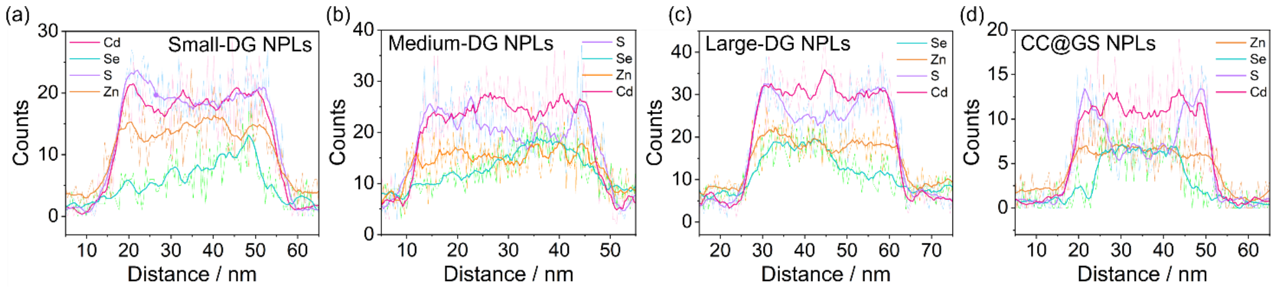

Figure S6. EDS line-scan analysis of an individual (a) small-DG NPL, (b) medium-DG NPL, (c) large-DG NPL, and (d) CC@GS NPL.

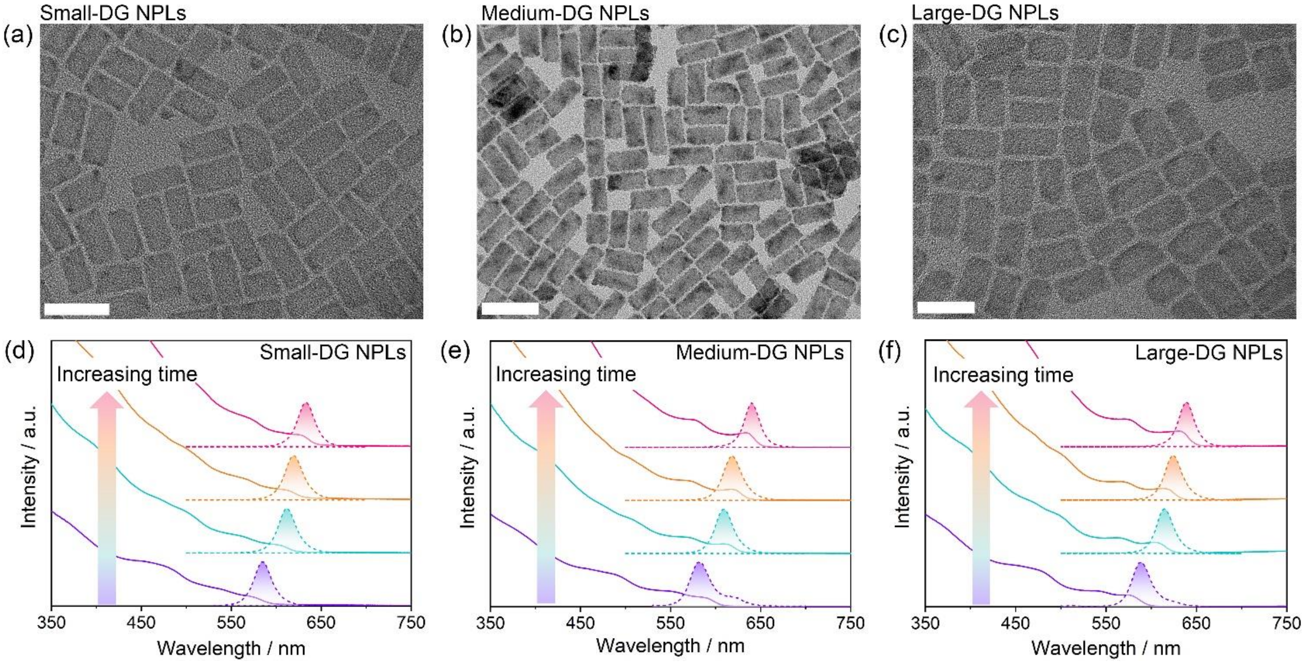

Figure S7. TEM images of the (a) small-, (b) medium-, and (c) large-DG NPLs (scale bar: 50 nm). The evolutions of the absorption and emission spectra of the (d) small-, (e) medium-, and (f) large-DG NPLs, monitored by taking small aliquots from the reaction solutions during the shell growth.

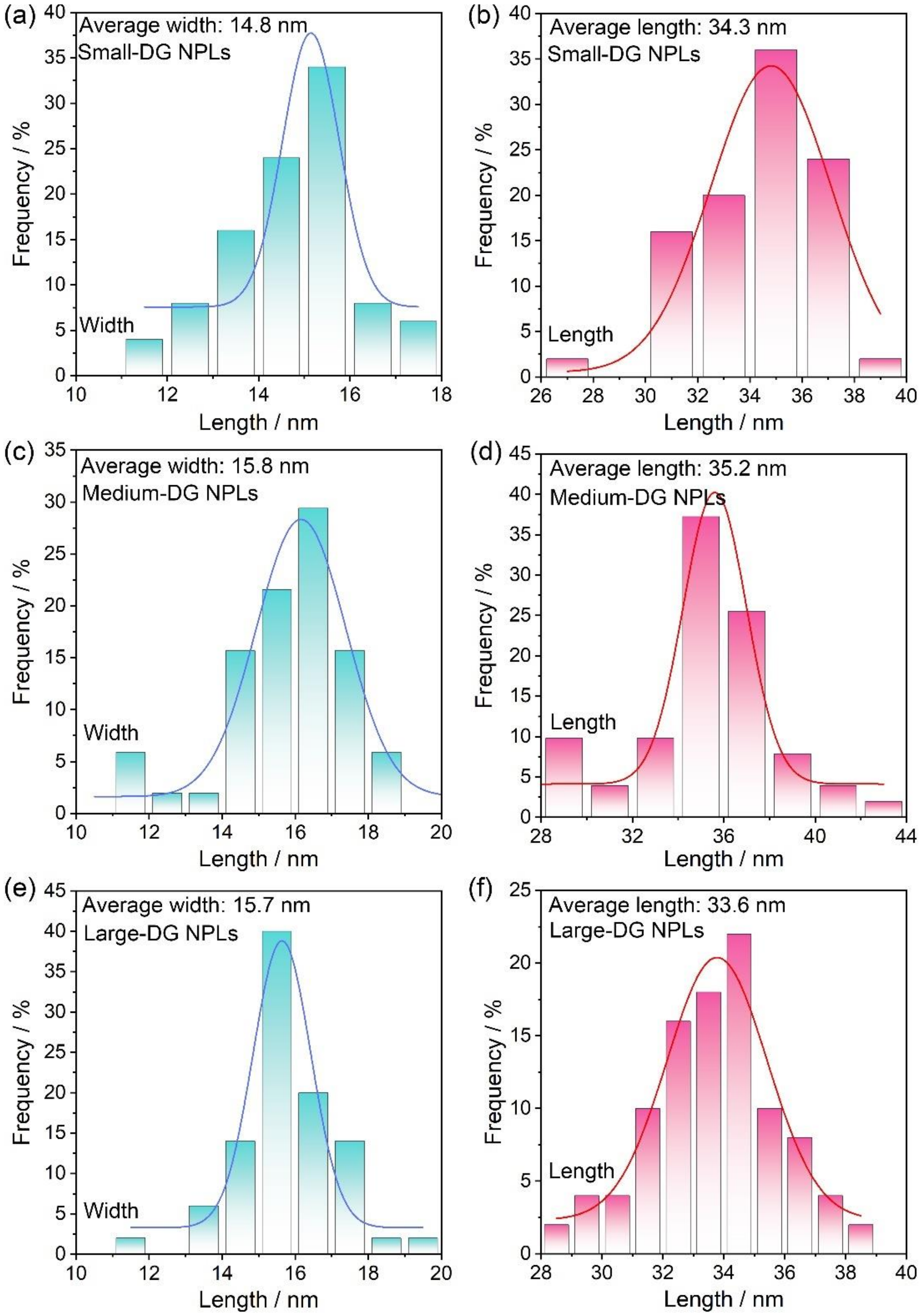

Figure S8. Size distributions of the width and length dimensions of (a,b) small-DG NPLs, (c,d) medium-DG NPLs, and (e,f) large-DG NPLs, respectively. The above results indicate that DG NPLs containing CdSe recombination centers of varying sizes exhibit comparable overall length and width dimensions.

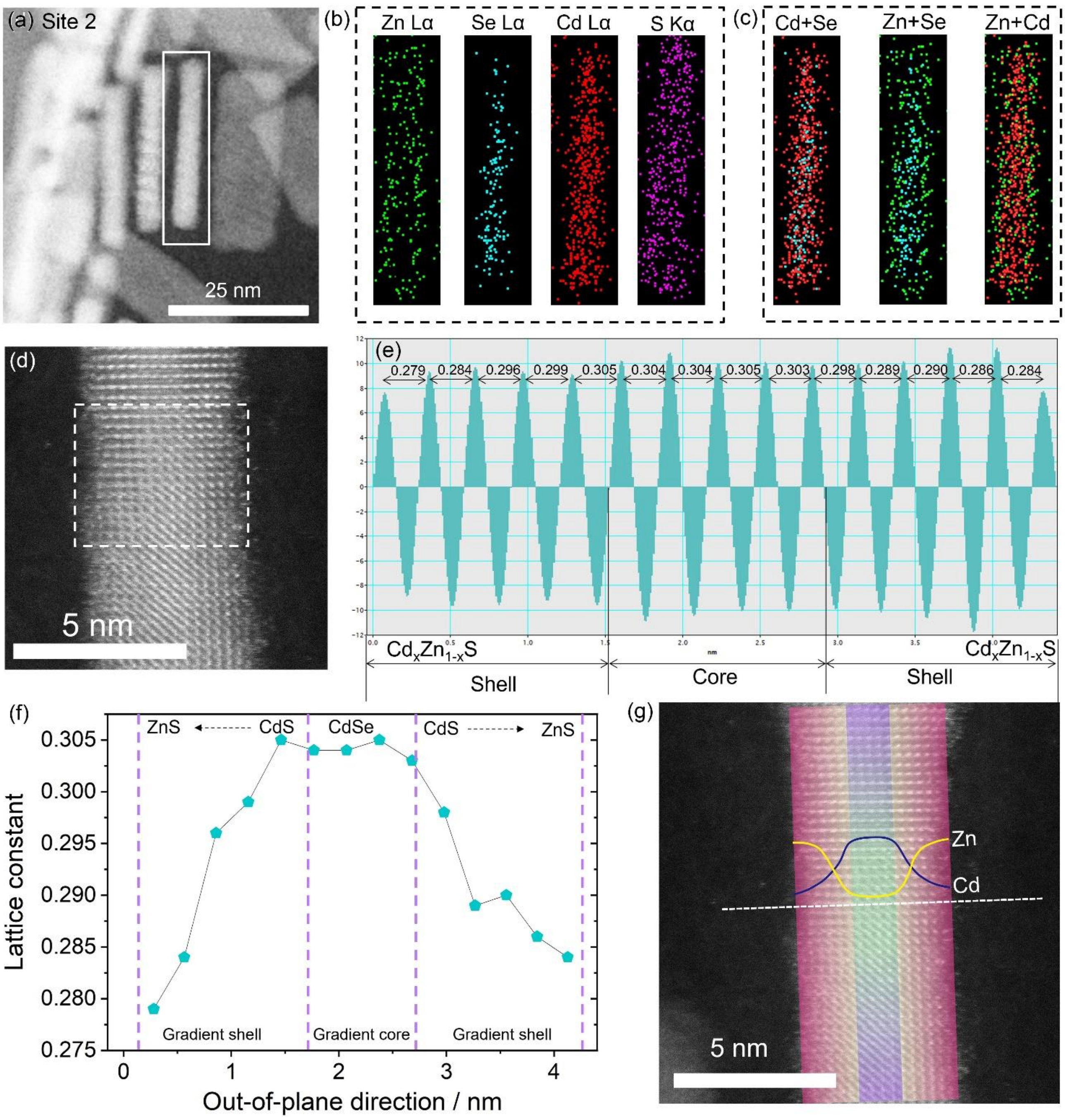

Figure S9. (a-c) Cross-sectional EDS mapping analysis of an individual DG NPL. (d) High-resolution STEM image showing the cross-section of an individual DG NPL. (e-f) Fourier analysis of lattice periodicity along the NPL thickness direction, indicating a gradual decrease in the lattice constant from the center of the NPL to the outer shell. (g) Schematic illustrations of the distributions of Zn and Cd on the cross-section of DG NPLs.

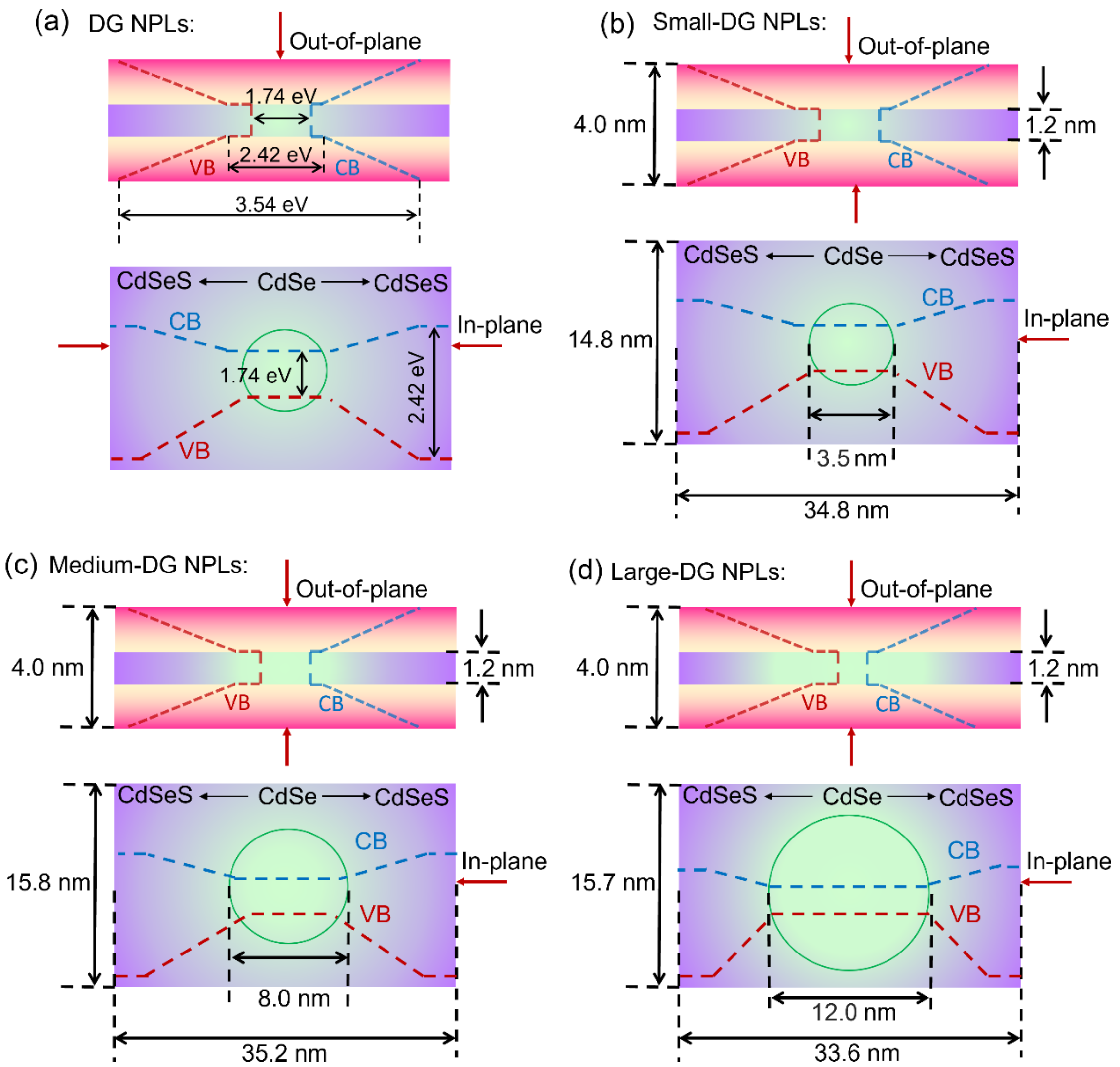

Figure S10. (a) Schematic band diagrams for both the in-plane and out-of-plane directions of DG NPLs. Please note that the bandgaps shown here are referenced according to the values of their bulk counterparts. Schematic illustrations of the band alignment structures of DG NPLs with (b) small, (c) medium, and (d) large CdSe sizes, respectively.

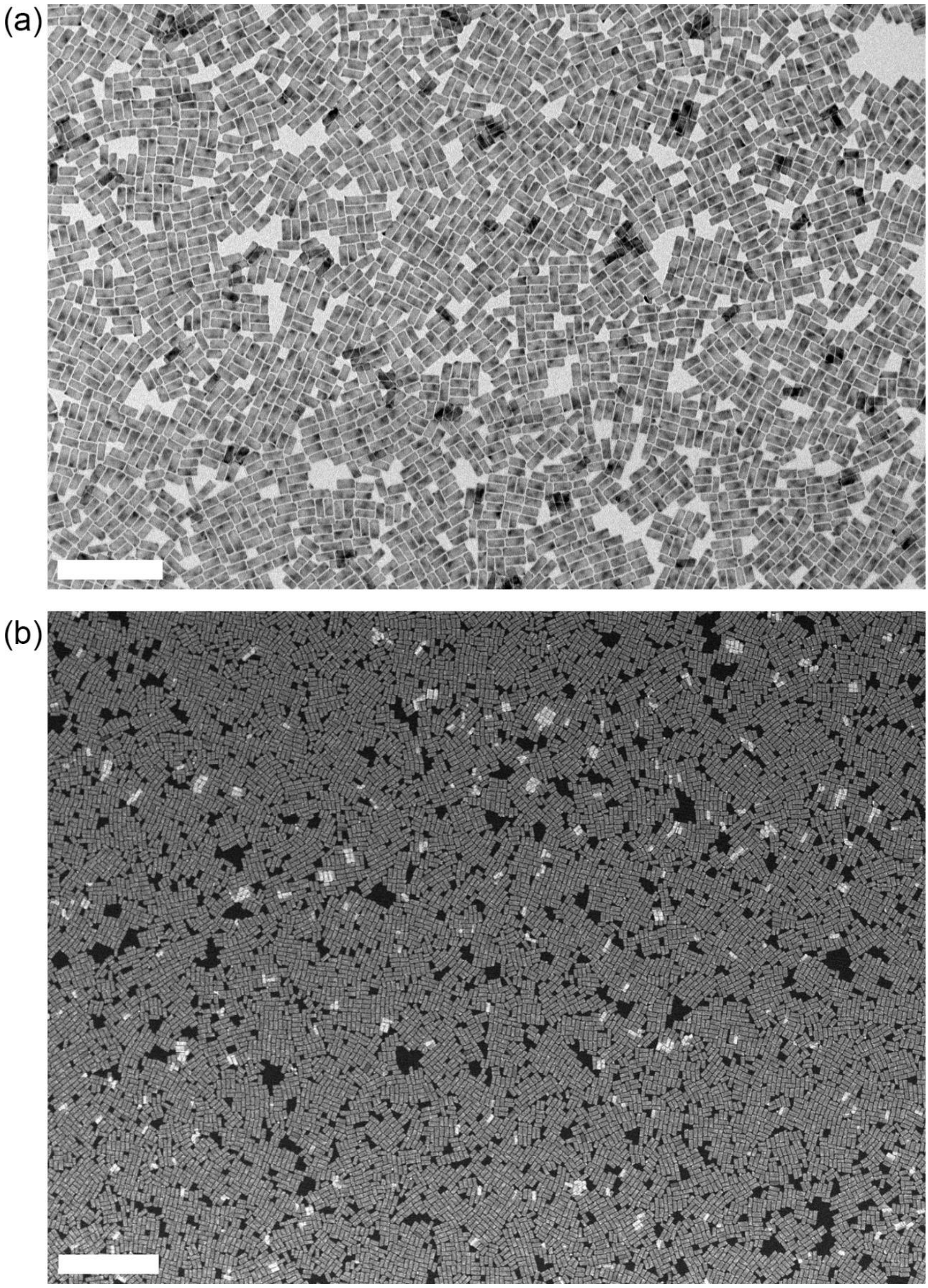

Figure S11. Representative large-area (a) TEM (scale bar: 200 nm) and (b) HAADF-STEM (scale bar: 400 nm) images of medium-DG NPLs, demonstrating the resulting DG NPLs after shell growth exhibit excellent uniformity in both lateral size and thickness.

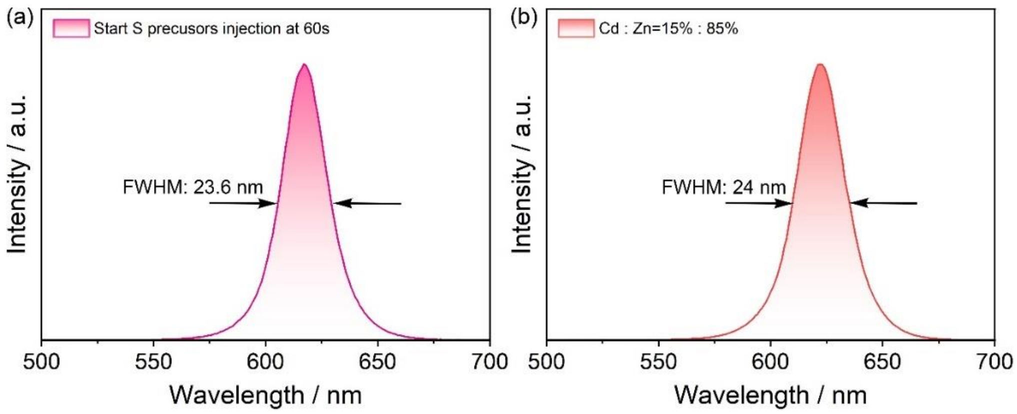

Figure S12. PL spectra of the resulting DG NPLs (a) using gradient cores with an earlier injection time of S precursors at 60 s and (b) by increasing the molar ratio of cations from Cd : Zn=25% : 75% to Cd : Zn=15% : 85% during the shell growth.

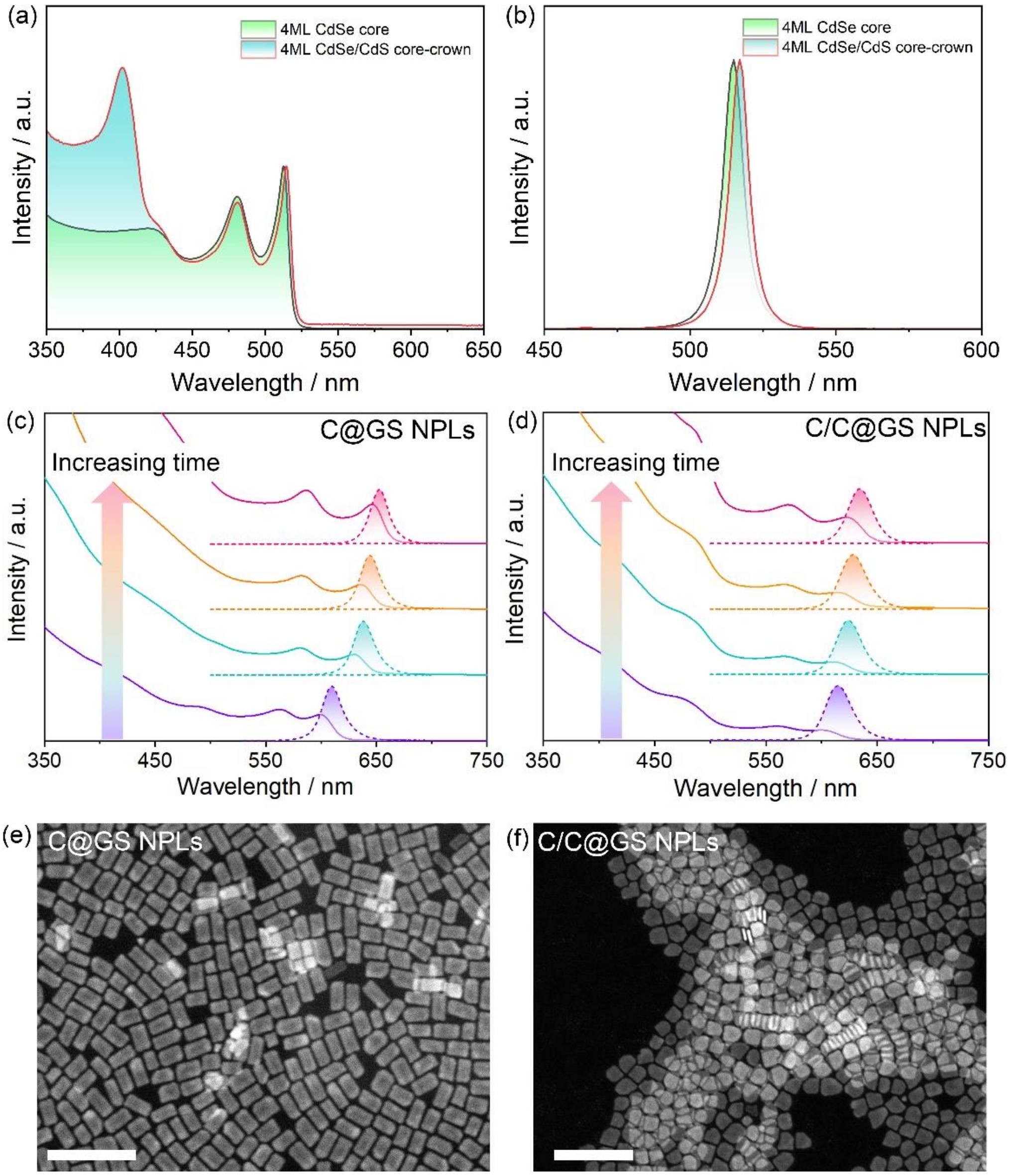

Figure S13. (a) Absorption and (b) PL spectra of the 4ML CdSe cores and 4ML CdSe/CdS core/crown

NPLs using the conventional growth methods. The evolutions of the absorption and emission spectra of the (c) C@GS NPLs and (d) C/C@GS NPLs, monitored by taking small aliquots from the reaction solution during the shell growth. HAADF-STEM images of the resulting (e) C@GS NPLs and (f) C/C@GS NPLs after shell growth (scale bar: 200 nm). These results are highly consistent with the previous reports of core-shell structured NPLs. [3, 8, 9]

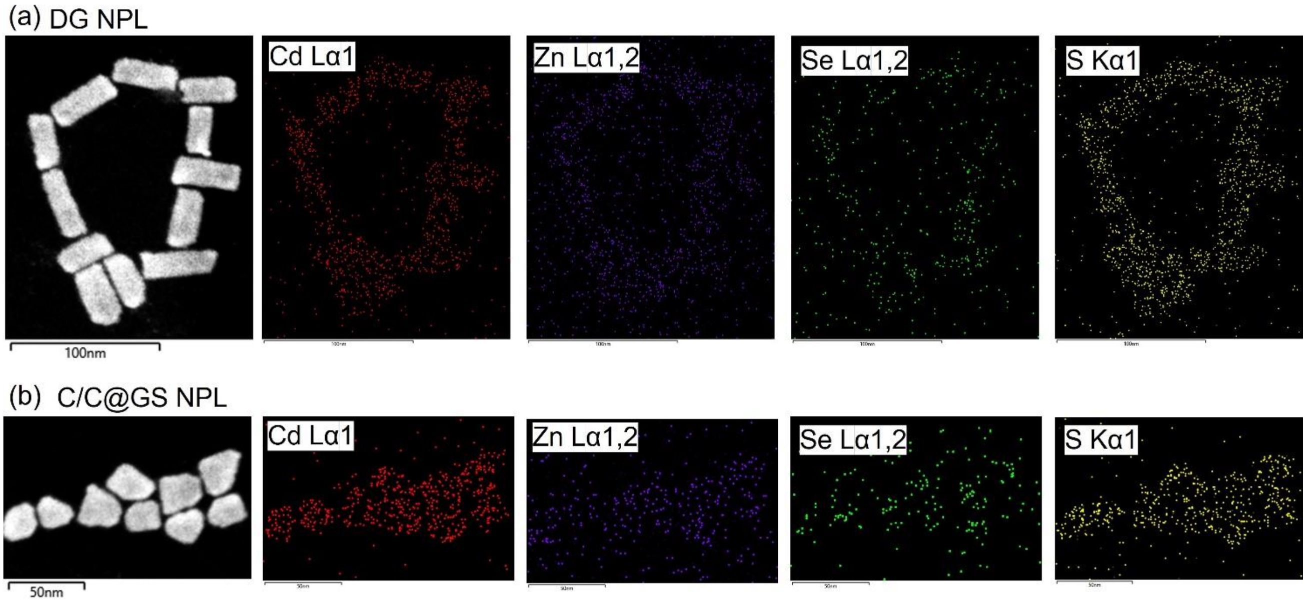

Figure S14. EDS mapping results of the (a) DG NPLs and (b) C/C@GS NPLs.

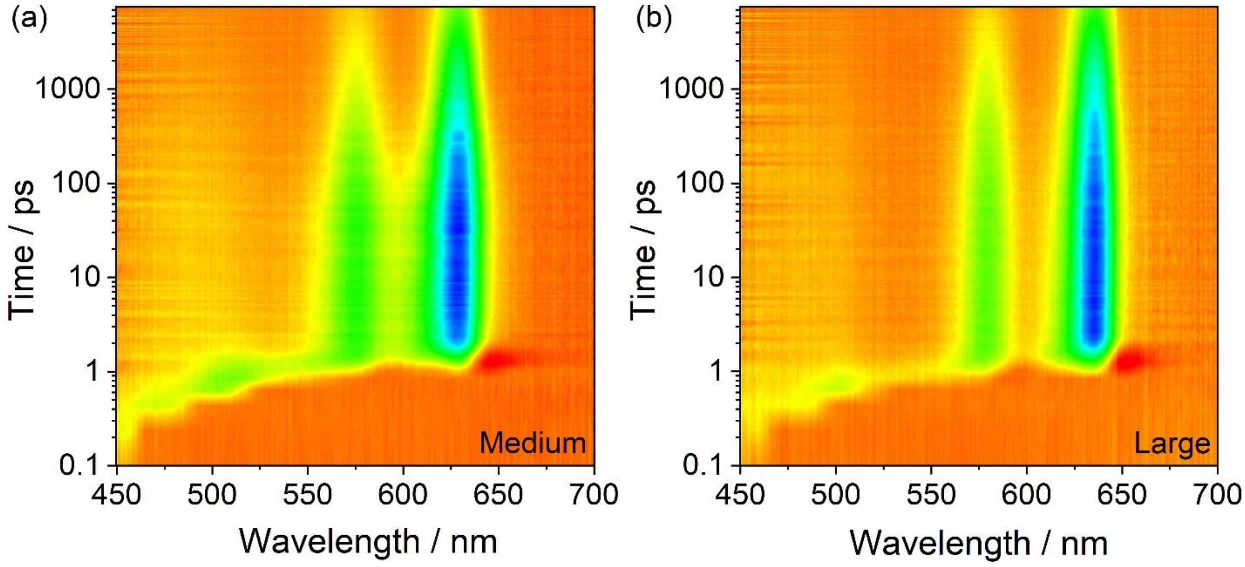

Figure S15. Transient absorption (TA) spectroscopies of (a) medium-DG NPLs and (b) large-DG NPLs. Similar to the results observed for small-DG NPLs, medium-, and large-DG NPLs also demonstrate highly efficient and ultrafast carrier transfer moving towards CdSe recombination center, with almost no trapped excitons in the $CdSe_xS_{1-x}$ region (450-550 nm).

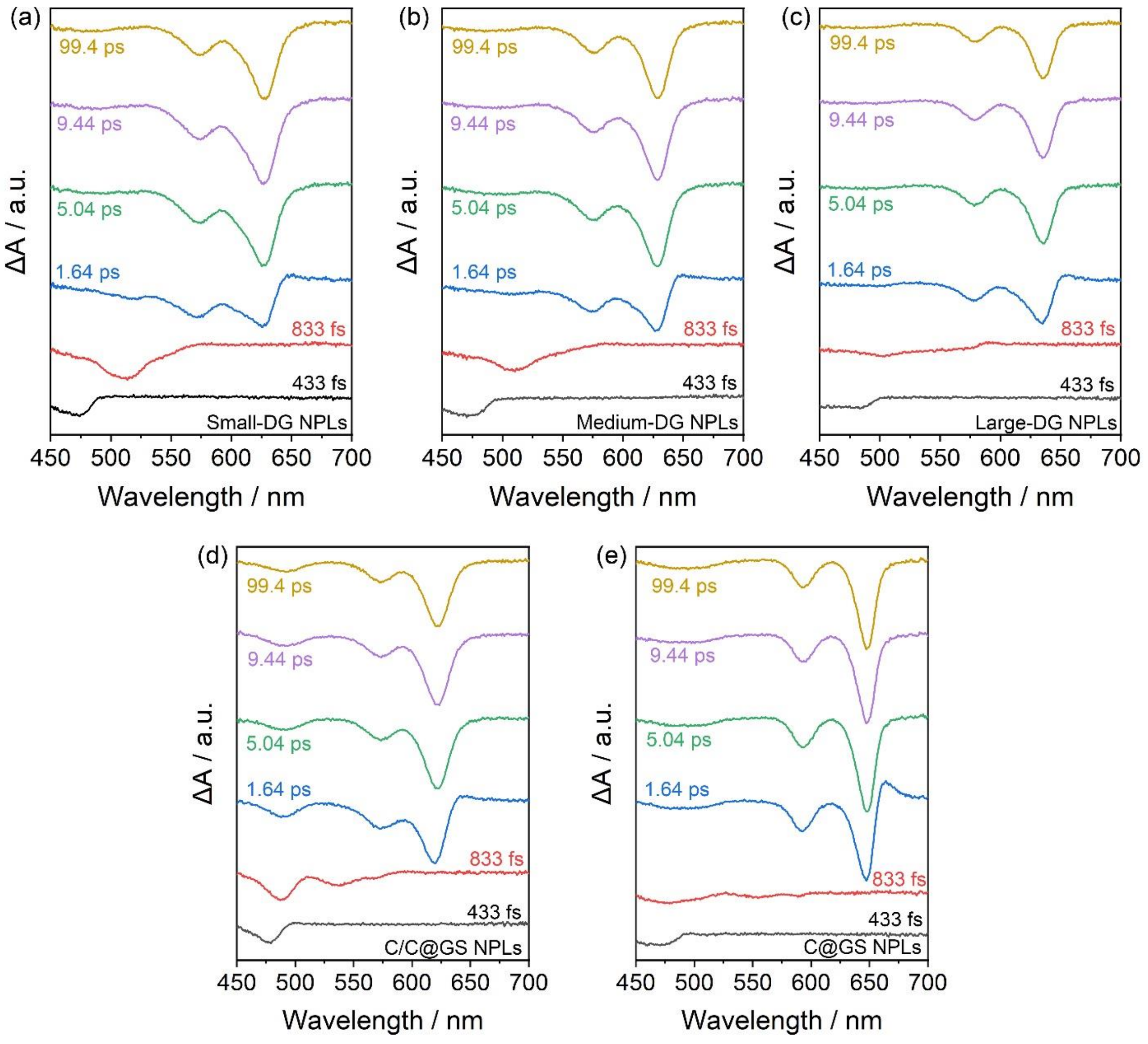

Figure S16. TA spectra of the (a) small-DG NPLs, (b) medium-DG NPLs, (c) large-DG NPLs, (d) C/C@GS NPLs, and (e) C@GS NPLs, reveal the evolution of ΔA at different decay intervals. As the relative size of CdSe become smaller, the bleach singles at 450-550 nm, corresponding to the excitons generated in the $CdSe_xS_{1-x}$ region, become stronger, reaching their maximum within the initial 833 fs. Afterward, the bleach singles of the light and heavy holes of CdSe become more intensive, accompanied by a fading of the 450-550 nm bleach single. The above results indicates that, at a consistent overall size of the DG NPLs, the smaller the size of the CdSe recombination center, the higher the exciton concentration ultimately achieved at the center. For conventional C/C@GS NPLs, controlling the concentration of the excitons in the recombination center is difficult, as the separation and purification of sub-10 nm 4ML CdSe cores are difficult to achieve synthetically. Furthermore, it can be observed that even after 99.4 ps, there is still a relatively strong bleach signal within the 450-550 nm range. This suggests that a large portion of excitons is trapped at the interface between CdS and CdSe, preventing the quantum efficiency from reaching near-unity. For C@GS NPLs, exciton generation and recombination are highly spatial overlapped within CdSe, leading to a significant self-absorption issue.

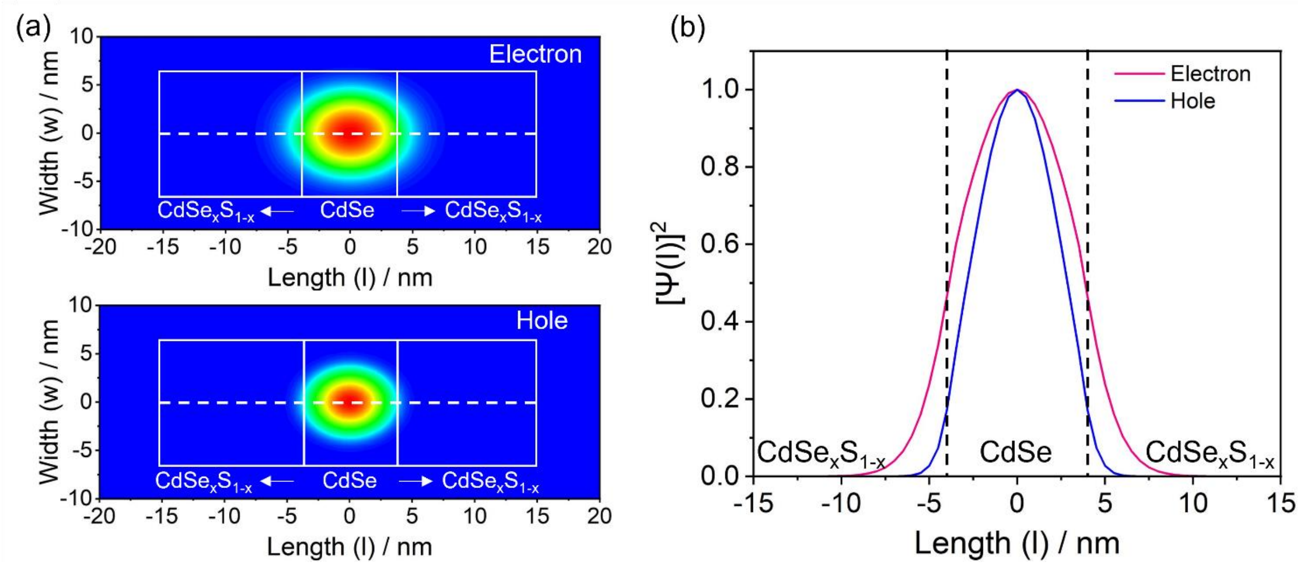

Figure S17. Figure S17. (a) Probability density of the electron and the hole within medium-DG NPLs. (b) The overlap of electrons and hole wavefunctions along the white dash line (w=0) in (a). The simulation method is referenced from our previous publication.[10]

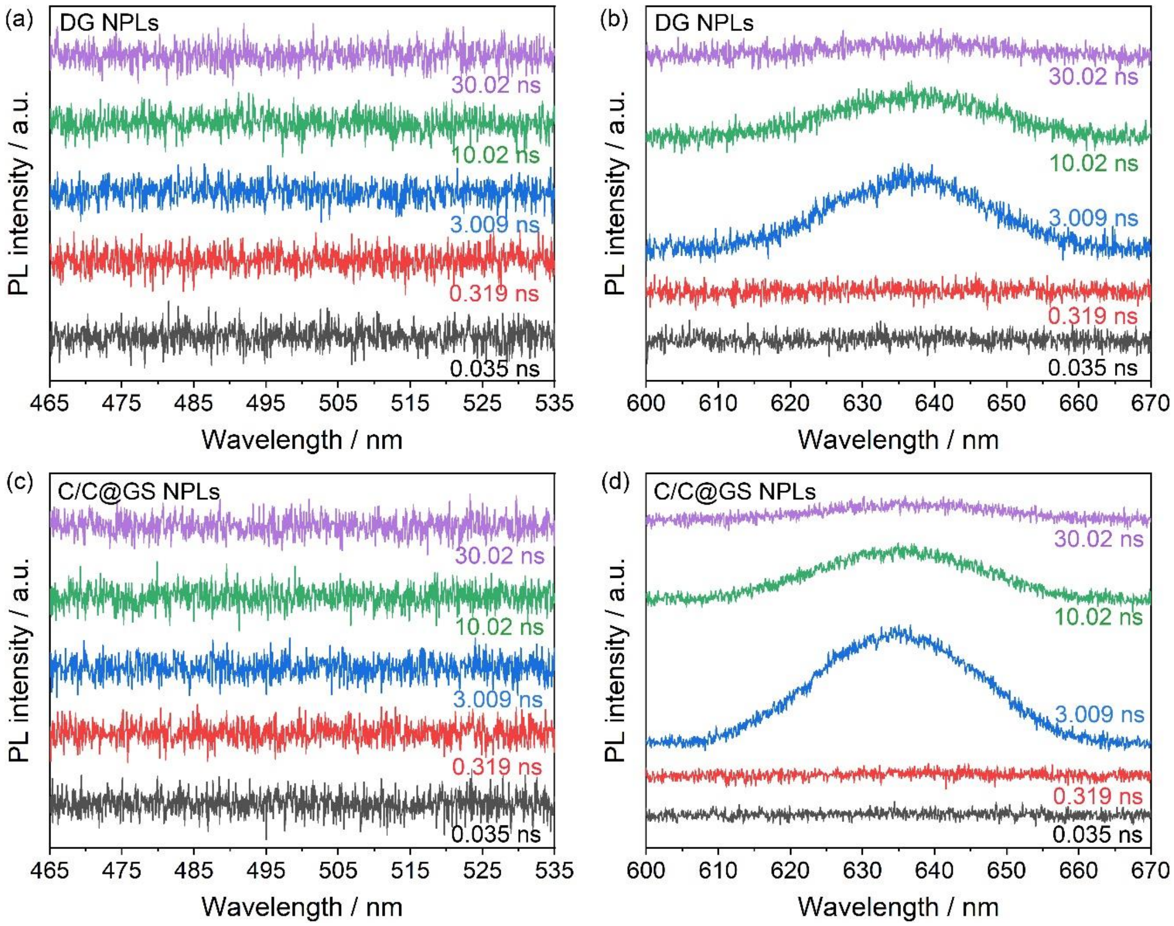

Figure S18. Time-resolved PL spectra of (a,b) DG NPLs and (c,d) C/C@GS NPLs at different decay intervals. These results demonstrate that all the emitted photons originate from exciton recombination

within the CdSe regions. Moreover, it also confirmed that the slow decay observed in the 450-550 nm range in the TA spectra of C/C@GS NPLs can be attributed to non-radiative trap states.

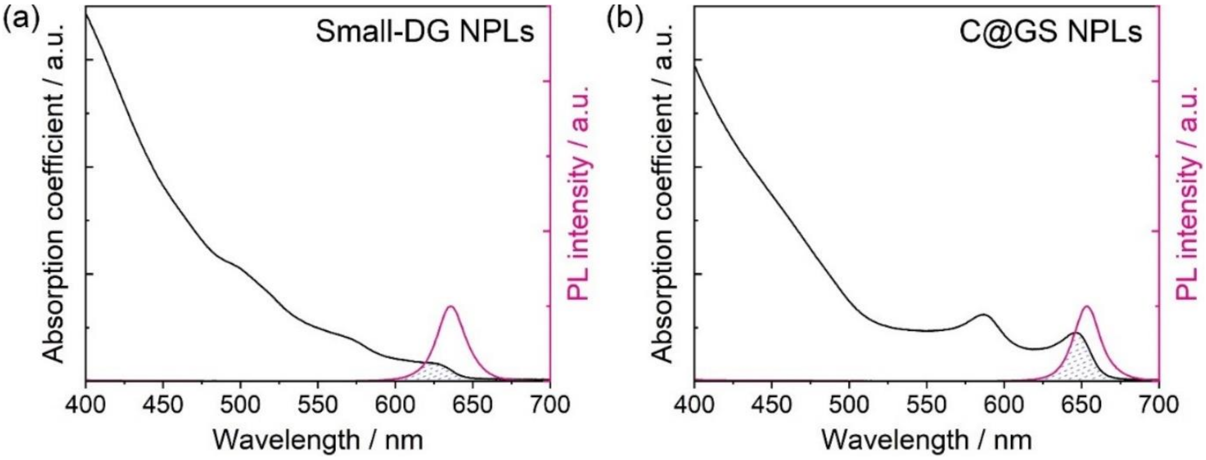

Figure S19. Absorption coefficient and PL spectra of (a) small-DG NPLs and (b) C@GS NPLs.

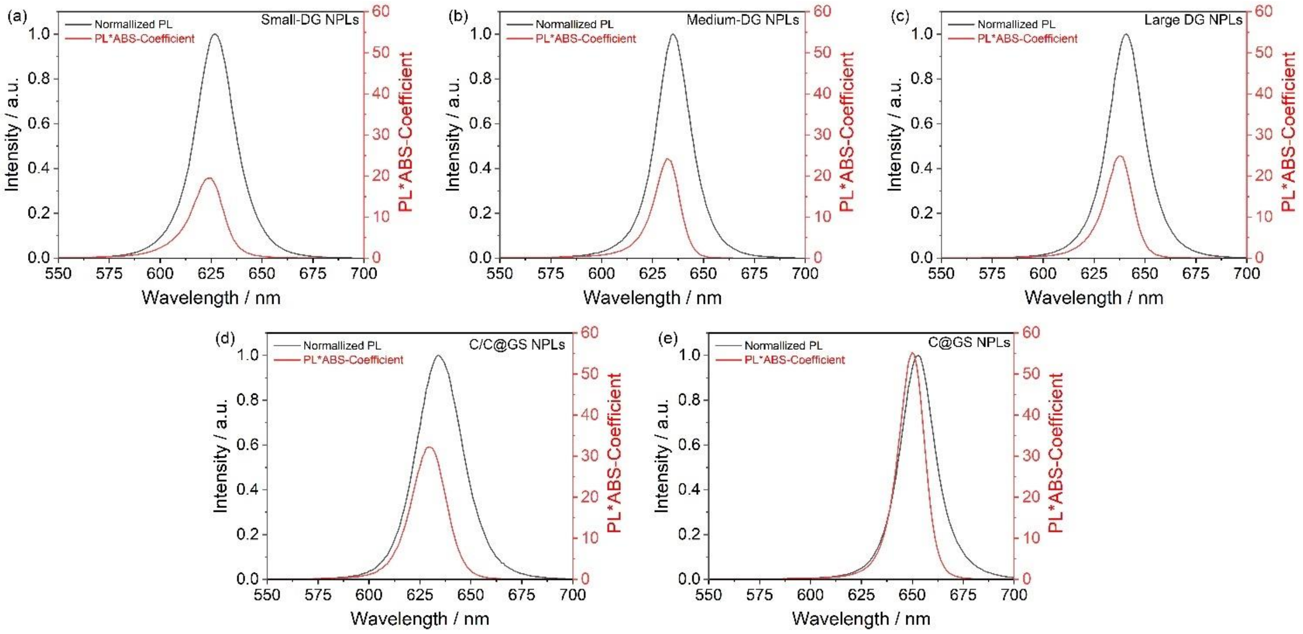

Figure S20. $S_{PL}(\lambda)\alpha(\lambda)$ spectra (red curves) and normalized PL spectra (black curves) of (a) small-DG NPLs, (b) medium-DG NPLs, (c) large-DG NPLs, (d) C/C@GS NPLs, and (e) C@GS NPLs.

The relative self-absorption coefficients of different samples are calculated and compared with each other according to the literature. [11] Specifically, the overlap between absorption coefficient spectra and PL spectra, as depicted in **Figure S11** using Small-DG NPLs and C@GS NPLs as examples, can be quantified as self-absorption coefficient α_s, according to **Equation 1**. Here $S_{PL}(\lambda)$ and $\alpha(\lambda)$ refer to PL spectra and absorption coefficient spectra, respectively.

$$\alpha_s = \frac{\int S_{PL}(\lambda)\alpha(\lambda)\,d\lambda}{\int S_{PL}(\lambda)\,d\lambda} \quad \textbf{(Equation 1)}$$

The calculated $S_{PL}(\lambda)\alpha(\lambda)$ in 550-700 nm for all samples are shown in the red curves in **Figure S12**, in comparison to their corresponding normalized PL spectra. It can be clearly seen that the higher overlap between absorption coefficient and PL spectra, the greater the weight of $S_{PL}(\lambda)\alpha(\lambda)$ in the PL spectra. Finally, α_s of different samples are calculated and compared with C@GS NPLs according to Equation 1 based on the data in **Figure S12**.

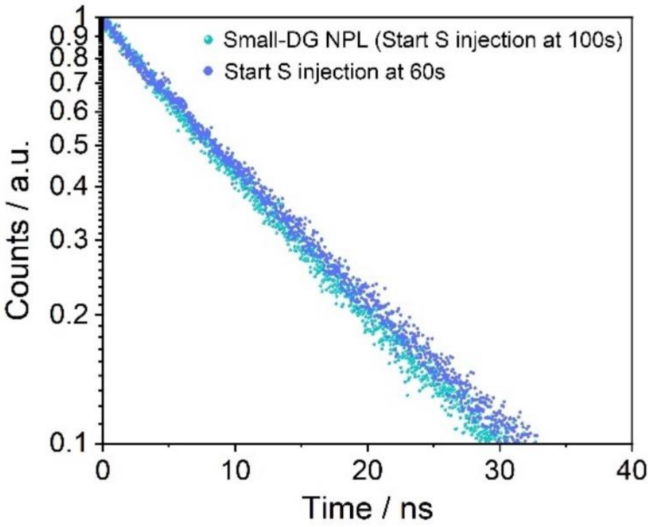

Figure S21. PL decay curves of small-DG NPL (start S precursors injection at 100 s, cyan dots) and DG NPLs with even smaller CdSe recombination center (start S precursors injection at 60 s, bule dots).

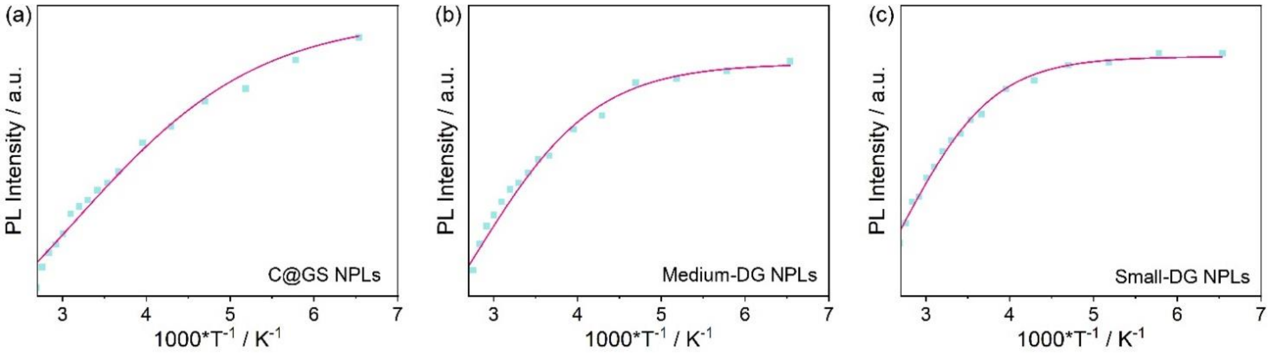

Figure S22. Integrated PL intensity as a function of temperature for (a) C@GS NPLs, (b) medium-DG NPLs, and (c) small-DG NPLs. The experimental data (cyan solid dots) are well-fitted by using **Equation 2** (magenta curves), as indicated by the literature.[12] The obtained E_b for C@GS NPLs, medium-DG NPLs, and small-DG NPLs is 78.2, 121.7, and 158.9 meV, respectively.

$$I(T) = \frac{I_0}{1+Ae^{(-E_b/k_BT)}} \quad \textbf{(Equation 2)}$$

Here I_0 refers to the PL intensity at 0 K, k_B is the Boltzmann constant and E_b is the exciton

binding energy.

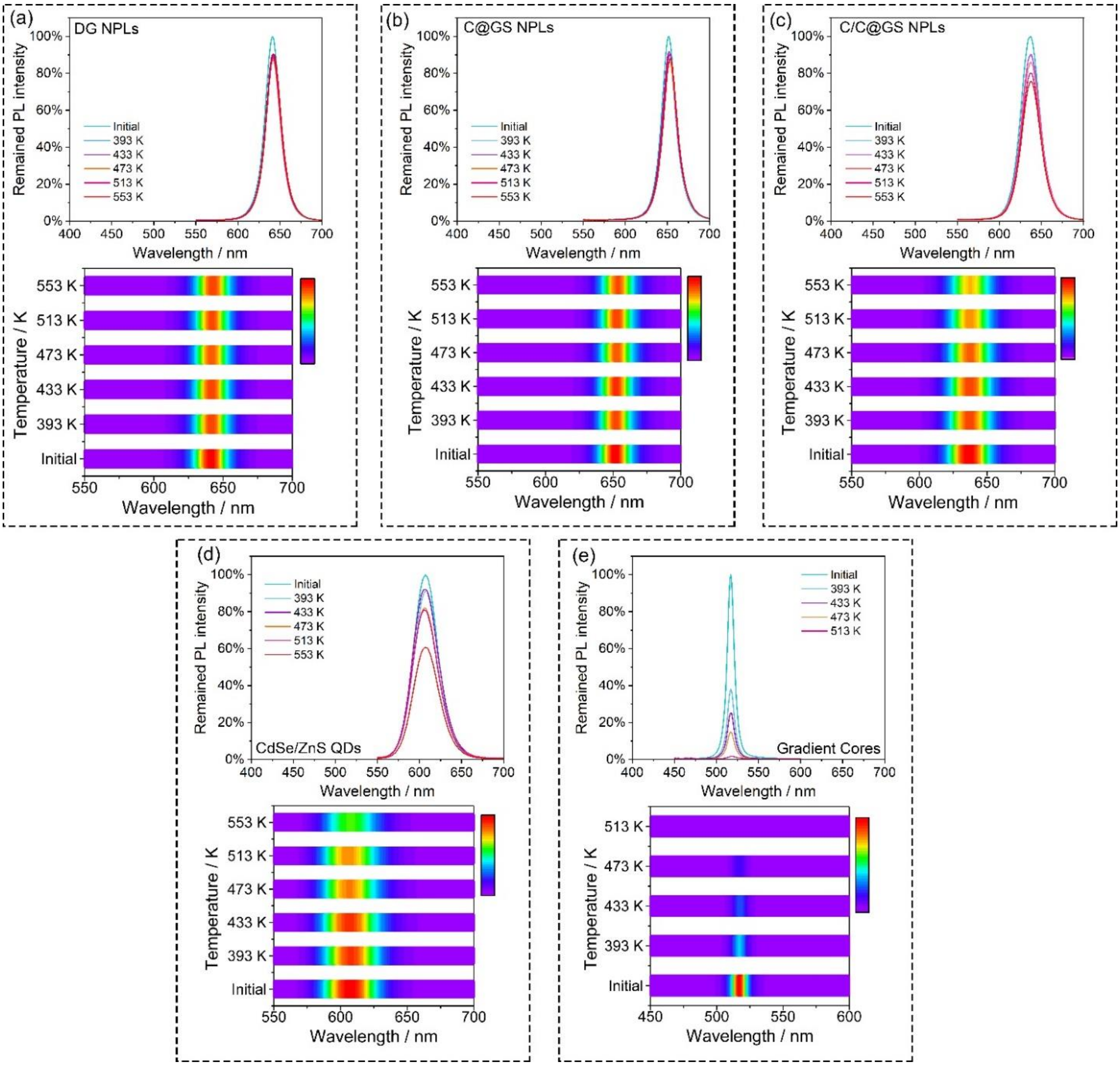

Figure S23. Thermal stability measurements of (a) DG NPLs, (b) C@GS NPLs, (c) C/C@GS NPLs, (d) CdSe@ZnS QDs, and (e) gradient cores by baking the samples at different elevated temperatures in the glove box for 10 min. The PL spectra of the samples were measured after the samples were cooled to room temperature.

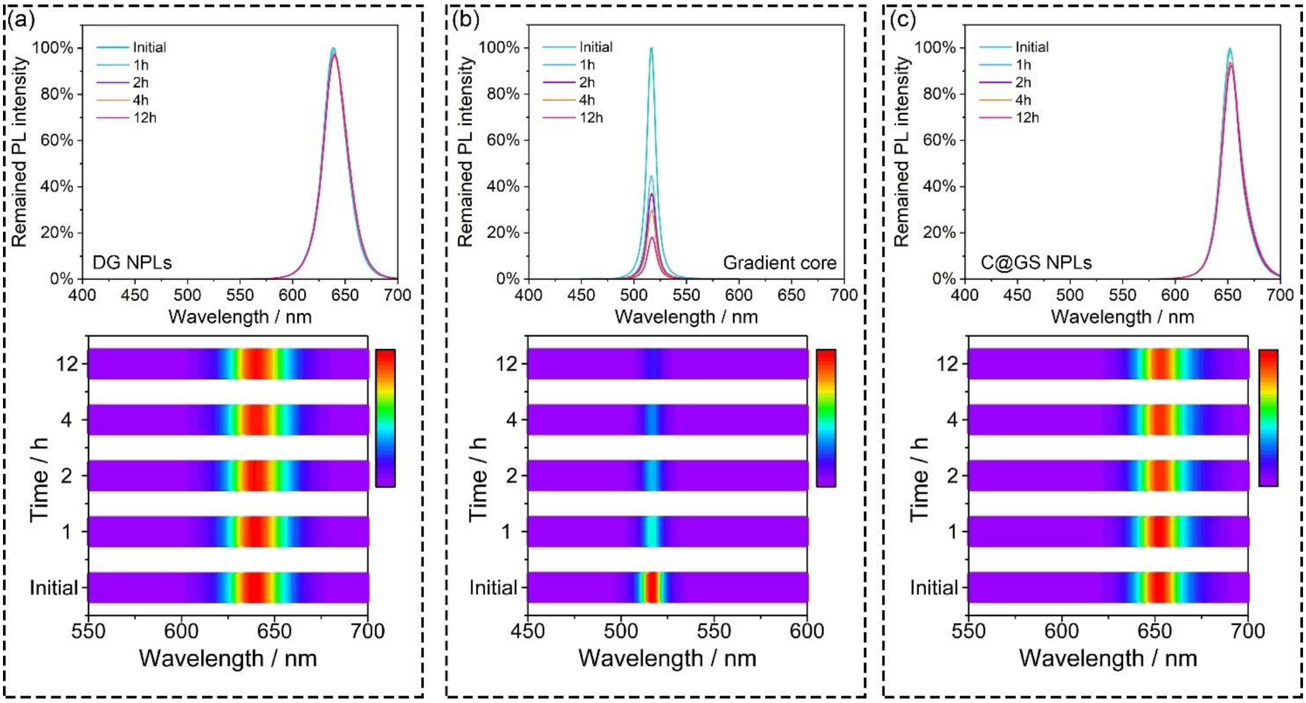

Figure S24. Long-term thermal stability measurements of (a) DG NPLs, (b) gradient core NPLs, and (c) C@GS NPLs by baking the samples in the air at 353 K. The PL spectra of the samples were measured at different time intervals during the baking process after the samples were cooled to room temperature.

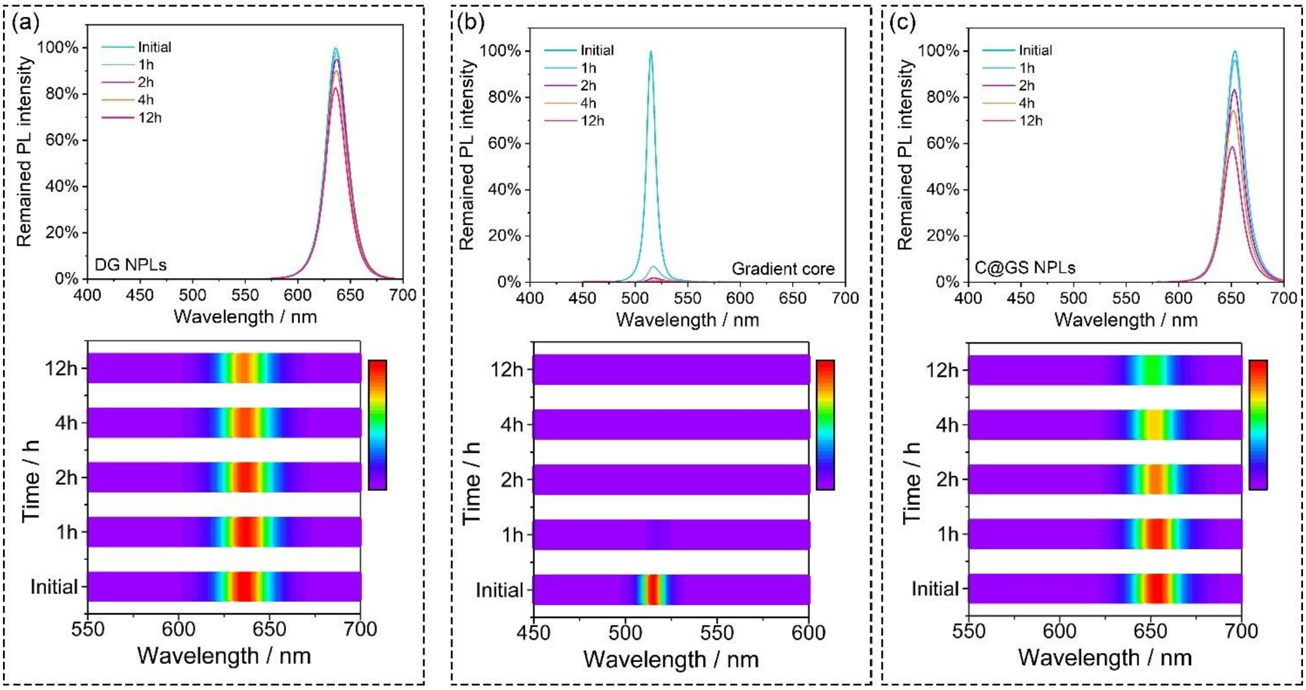

Figure S25. Long-term thermal stability measurements of (a) DG NPLs, (b) gradient core NPLs, and (c) C@GS NPLs by baking the samples in the air at 393 K. The PL spectra of the samples were measured at different time intervals during the baking process after the samples were cooled to room temperature.

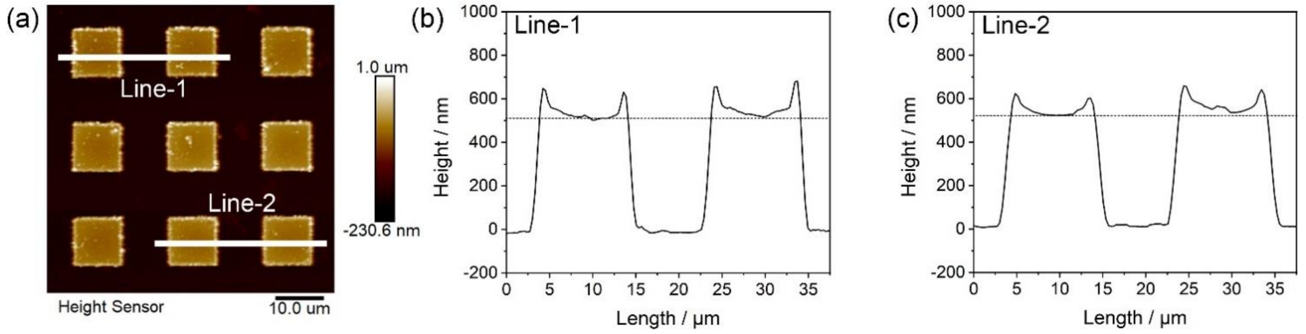

Figure S26. (a) AFM image of the DG NPLs pixels. The surface profiles of the DG NPLs pattern for (b) line-1 and (c) line-2 in the AFM image. The results show the large thickness of ~500 nm for the resulting NPLs pattern.

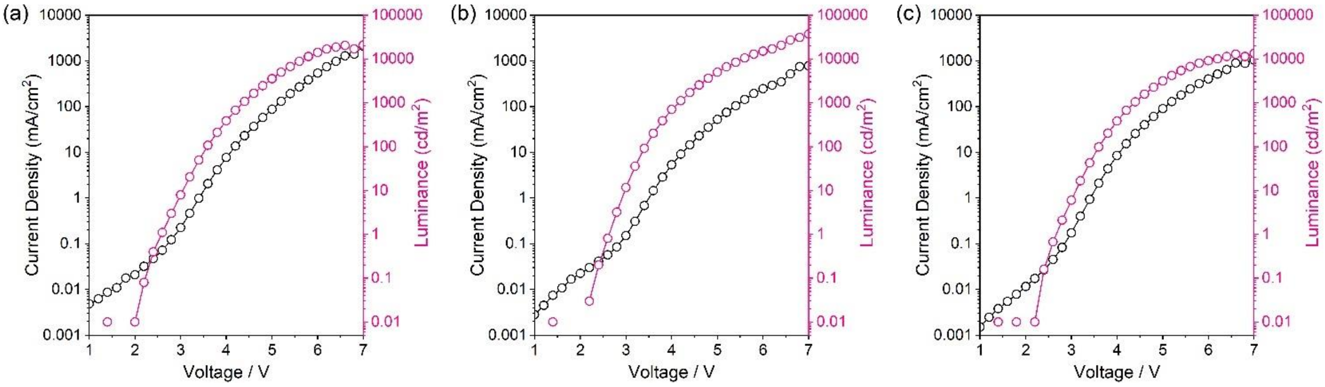

Figure S27. Current density and luminance of (a) small-DG NPLs, (b) medium-DG NPLs, and (c) C@GS NPLs at different applied voltages.

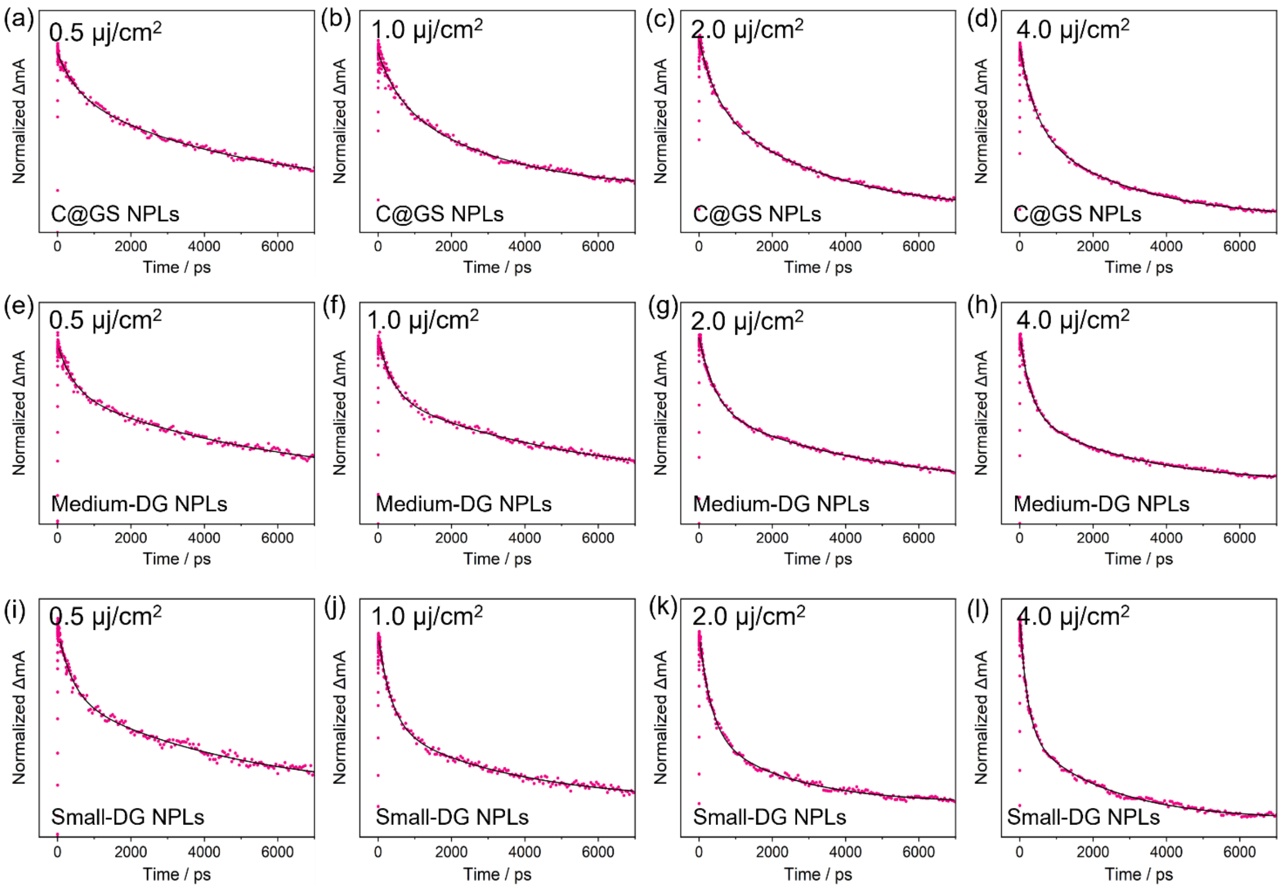

Figure S28. Exitons decay kinetics of (a-d) C@GS NPLs, (e-h) medium-DG NPLs, and (i-l) small-DG NPLs at their first excitonic peaks under different pump fluences.

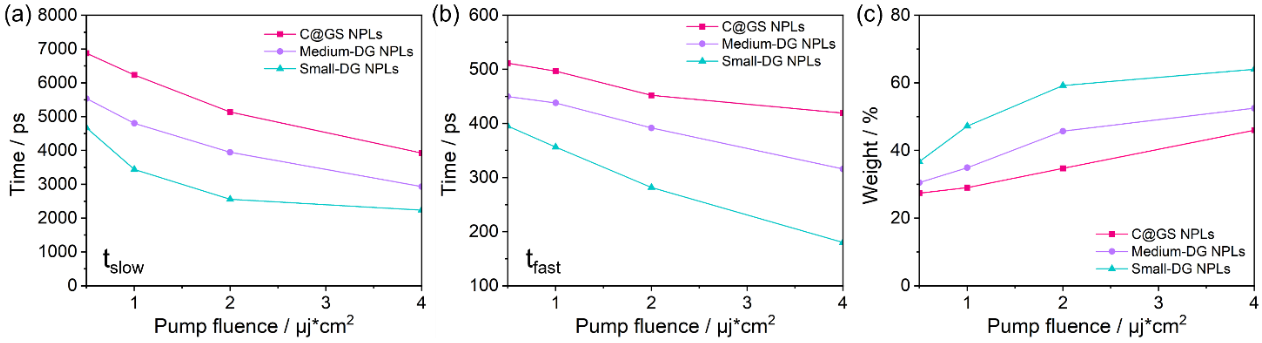

Figure S29. Summary of the lifetimes of the (a) slow-decay component, (b) fast-decay component, and (c) weight of the fast-decay component by fitting the power-dependent exciton kinetics in **Figure S28** using a bi-exponential fitting for C@GS NPLs, medium-DG NPLs, and small-DG NPLs, respectively.

Reference

1. Altintas Y*, et al.* Highly Stable, Near-Unity Efficiency Atomically Flat Semiconductor Nanocrystals of CdSe/ZnS Hetero-Nanoplatelets Enabled by ZnS-Shell Hot-Injection Growth. *Small* **15**, 1804854 (2019).

2. Bertrand GH, Polovitsyn A, Christodoulou S, Khan AH, Moreels I. Shape control of zincblende CdSe nanoplatelets. *ChemComm* **52**, 11975-11978 (2016).

3. Taghipour N*, et al.* Sub-single exciton optical gain threshold in colloidal semiconductor quantum wells with gradient alloy shelling. *Nat. Commun.* **11**, 3305 (2020).

4. Lim J, Jun S, Jang E, Baik H, Kim H, Cho J. Preparation of highly luminescent nanocrystals and their application to light-emitting diodes. *Adv. Mater.* **19**, 1927-1932 (2007).

5. Hu S*, et al.* High-performance deep red colloidal quantum well light-emitting diodes enabled by the understanding of charge dynamics. *ACS Nano* **16**, 10840-10851 (2022).

6. Gheshlaghi N*, et al.* Self-resonant microlasers of colloidal quantum wells constructed by direct deep patterning. *Nano Lett.* **21**, 4598-4605 (2021).

7. Yoon, D.-E.; Lee, J.; Yeo, H.; Ryou, J.; Lee, Y. K.; Kim, Y.-H.; Lee, D. C. Atomistics of asymmetric lateral growth of colloidal zincblende CdSe nanoplatelets. *Chemistry of Materials* **2021**, *33* (12), 4813-4820.

8. Samadi Khoshkhoo M, Prudnikau A, Chashmejahanbin MR, Helbig R, Lesnyak V, Cuniberti G. Multicolor patterning of 2D semiconductor nanoplatelets. *ACS Nano* **15**, 17623-17634 (2021).

9. Shabani F*, et al.* Deep-Red-Emitting Colloidal Quantum Well Light-Emitting Diodes Enabled through a Complex Design of Core/Crown/Double Shell Heterostructure. *Small* **18**, 2106115

(2022).

10. Shabani, F.; Martinez, P. L. H.; Shermet, N.; Korkut, H.; Sarpkaya, I.; Dehghanpour Baruj, H.; Delikanli, S.; Isik, F.; Durmusoglu, E. G.; Demir, H. V. Gradient Type-II CdSe/CdSeTe/CdTe Core/Crown/Crown Heteronanoplatelets with Asymmetric Shape and Disproportional Excitonic Properties. *Small* **2023**, *19* (11), 2205729.

11. Klimov VI, Baker TA, Lim J, Velizhanin KA, McDaniel H. Quality factor of luminescent solar concentrators and practical concentration limits attainable with semiconductor quantum dots. *ACS Photonics* **3**, 1138-1148 (2016).

12. Li F*, et al.* Enhancing exciton binding energy and photoluminescence of formamidinium lead bromide by reducing its dimensions to 2D nanoplates for producing efficient light emitting diodes. *Nanoscale* **10**, 20611-20617 (2018).
